# Reversible redox controlled acids for cationic ring-opening polymerization†

**DOI:** 10.1039/d1sc03011f

**Published:** 2021-07-06

**Authors:** Michael J. Supej, Elizabeth A. McLoughlin, Jesse H. Hsu, Brett P. Fors

**Affiliations:** Department of Chemistry and Chemical Biology, Cornell University Ithaca New York 14853 USA bpf46@cornell.edu

## Abstract

Advancements in externally controlled polymerization methodologies have enabled the synthesis of novel polymeric structures and architectures, and they have been pivotal to the development of new photocontrolled lithographic and 3D printing technologies. In particular, the development of externally controlled ring-opening polymerization (ROP) methodologies is of great interest, as these methods provide access to novel biocompatible and biodegradable block polymer structures. Although ROPs mediated by photoacid generators have made significant contributions to the fields of lithography and microelectronics development, these methodologies rely upon catalysts with poor stability and thus poor temporal control. Herein, we report a class of ferrocene-derived acid catalysts whose acidity can be altered through reversible oxidation and reduction of the ferrocenyl moiety to chemically and electrochemically control the ROP of cyclic esters.

## Abstract 2

A class of ferrocene-derived acid catalysts whose acidity can be altered through reversible oxidation and reduction of the ferrocenyl moiety to chemically and electrochemically control the ROP of cyclic esters is reported.

## Graphical abstract

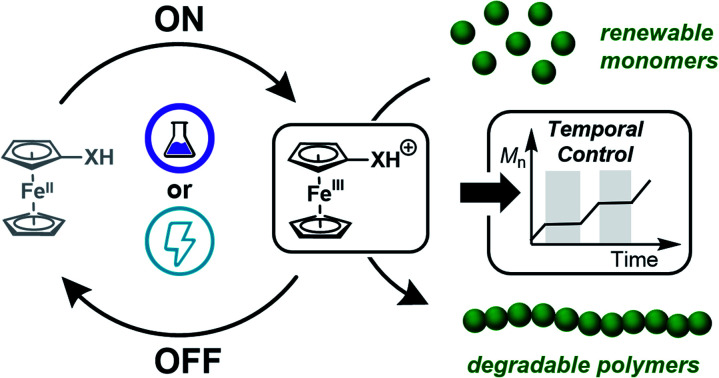

## Introduction

The physical properties and functionalities of polymeric materials are directly correlated to their structure and architecture.^1–3^ While living polymerization methodologies in conjunction with carefully chosen and timed monomer additions produce well-defined materials (*e.g.*, block copolymers^2^), the ability to control chain growth with an external stimulus could lead to many advanced structures and architectures with potentially interesting physical properties. These externally controlled polymerization methodologies rely on changes in chemical reactivity upon application of an external stimulus (chemical,^4–17^ electrochemical,^18–20^ photochemical,^21–30^ thermal,^31–33^ mechanochemical^34–37^), which precisely regulates the incorporation of monomers at a growing polymer chain end. In addition to promoting the synthesis of advanced structures and architectures,^30,45^ the spatiotemporal control afforded by these externally controlled polymerizations has enabled the development of new lithographic^38–42^ and 3D printing technologies.^43,44^

The development of methods for externally controlled ring-opening polymerization (ROP) of cyclic esters and carbonates is of particular interest as the polymers that are formed are biocompatible and degradable alternatives to petroleum-derived polyolefins. State-of-the-art methods for externally controlled ROP have involved the use of redox-switchable coordination-insertion catalysts, which have been pioneered by Byers^7,10–12,20^ and Diaconescu,^8,9,13–17^ or the use of photoacids, which induce polymerization through an activated monomer mechanism. Photoacid generators (PAGs)^46^ have been pivotal to the fields of lithography and microelectronics development; however, PAG-mediated polymerizations are not reversible and only provide temporal control over polymer initiation rather than chain growth. To overcome this challenge and develop a reversible photoacid, Boyer and Read de Alaniz have independently used merocyanine-based catalysts.^47,48^ However, slow thermal reversion of the spiropyran to the protonated merocyanine limits the degree of temporal control in these systems. Similarly, Hecht and Liao have each reported catalysts for photoswitchable ROP,^49,50^ but limitations related to catalytic efficiency and reversibility were encountered in these systems as well. On this basis, the discovery of an acid catalyst that can be reversibly activated by an external stimulus for ROP remains a challenge.

**Fig. 1:**
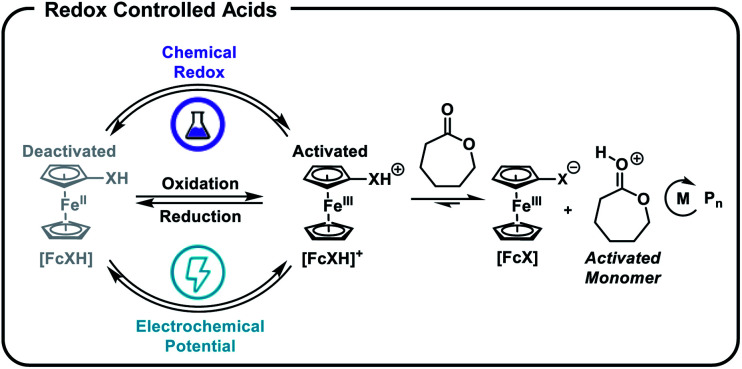
Ferrocenyl acids afford chemical and electrochemical control over the ring-opening polymerization (ROP) of cyclic esters.

We postulated that temporal control over acid-catalysed, cationic ROP could be achieved by designing a reversible, redox-controlled acid whose p*K*_a_ could be altered through changes in oxidation state.^51,52^ Specifically, by tethering ferrocene to an acidic functional group^53,54^ we envisaged a system where the p*K*_a_ would decrease upon oxidation from Fe(ii) to Fe(iii) and subsequently initiate ROP by an activated monomer mechanism (Fig. 1). Importantly, reduction of the ferrocenium species back to ferrocene would restore the original acidity of the molecule and deactivate the catalyst, affording reversible termination and thus temporal control over the polymerization.

## Results and discussion

To test our hypothesis, we examined the ROP of caprolactone (CL) catalysed by substituted ferrocenes in both the absence and presence of oxidant (Table 1). We selected benzyl alcohol (BnOH) as the initiator for its relatively high oxidation potential^55^ and its ability to provide an NMR label at the polymer chain end. Combining CL, BnOH, and either ferrocenyl phosphonic (**1a**), ferrocenyl (phenyl)phosphinic (**1b**), or ferrocenyl (phenyl)phosphonic (**1c**) acid showed no polymerization (see ESI Table S1,† entries 1–3). These results demonstrate that these acids in their neutral state are not competent catalysts for polymerization of CL. Significantly, addition of 1.2 equivalents of AgBF_4_ relative to the ferrocenyl acid in all three of these reactions efficiently initiated ROP and gave polymers with good agreement between theoretical and experimental number average molar masses (*M*_*n*_s) and low dispersity (*Đ*) values (Table 1, entries 1–3).^56^ It is worth noting that analogous reactivity was also observed in dichloromethane (DCM) (Table 1, entry 6) and the polymer obtained had a lower dispersity value. Furthermore, no polymerization was observed in the absence of ferrocenyl acid (Table 1, entry 7). In the absence of BnOH initiator, we observed 2% monomer conversion, which we attribute to the presence of trace water (Table 1, entry 9). The ferrocenyl carboxylic acid catalysts **2a** and **2b** showed little to no catalytic activity in either the presence or absence of oxidant (Table 1, entries 4–5; ESI Table S1,† entries 4–5), demonstrating that the identity of the acid is pivotal for efficient polymerization.

**Table 1:** Ferrocenyl acids for controlled cationic ring-opening polymerization of CL

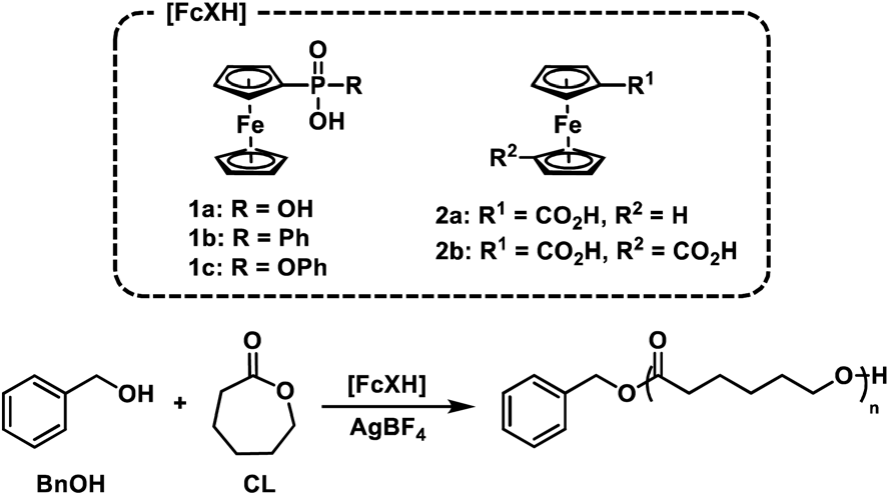						
Entry	Acid catalyst	[CL] : [BnOH] : [FcXH] : [Ox]	Conversion	*M*_*n*,Theo_^f^ (kg mol^−1^)	*M*_*n*,Exp_^g^ (kg mol^−1^)	*Đ*
1^a^	**1a**	100 : 1 : 1 : 1.2	52%	6.0	5.6	1.15
2^a^	**1b**	100 : 1 : 1 : 1.2	36%	4.1	3.2	1.14
3^a^	**1c**	100 : 1 : 1 : 1.2	45%	5.9	4.9	1.15
4^a^	**2a**	100 : 1 : 1 : 1.2	<5%	—	—	—
5^a^	**2b**	100 : 1 : 1 : 1.2	<5%	—	—	—
6^b^	**1b**	100 : 1 : 1 : 1.2	35%	4.1	4.0	1.07
7^b^	**1b**	100 : 1 : 1 : 0	0%	—	—	—
8^b^	**1b**	100 : 1 : 0 : 1.2	0%	—	—	—
9^b^	**1b**	100 : 0 : 1 : 1.2	2%	—	10.6	1.09
10^b^^,^^c^	**1b**	200 : 1 : 1 : 1.2	49%	11.3	10.9	1.26
11^b^	**1b**	100 : 1 : 2 : 2.4	65%	7.5	6.3	1.13
12^b^	**1b**	53 : 1 : 1 : 1.2	87%	5.3	4.2	1.09
13^b^	**1b**	26 : 1 : 1 : 1.2	>99%	3.0	2.3	1.09
14^b^	**1b**	11 : 1 : 1 : 1.2	>99%	1.3	1.3	1.05
15^d^	**1c**	51 : 1 : 0.5 : —	65%	2.8	3.8	1.08
16^d^	**1c**	51 : 1 : 0 : —	>99%	8.8	5.9	1.86
17^e^	**1c**	51 : 1 : 0.5 : —	0%	—	—	—

a [CL] = 9.1 M (neat), 1.0 mol% [FcXH], 1.2 mol% AgBF_4_, 23 °C.

b [CL] = 4.8 M (in DCM).

c CL was metered in at a rate of 0.1 mL per hour.

d [CL] = 4.98 M (in DCM), 0.2 M Bu_4_NBF_4_, 2.0 mA constant current, RVC anode, RVC cathode.

e [CL] = 4.98 M (in DCM), 0.2 M Bu_4_NBF_4_, no applied current, RVC anode, RVC cathode.

f *M*_*n*,Theo_ = [CL]/[BnOH] × MW_CL_ × conversion + MW_BnOH_.

g *M*_*n*,Exp_ determined by gel permeation chromatography with a multiangle light scattering (MALS) detector.

**Fig. 2:**
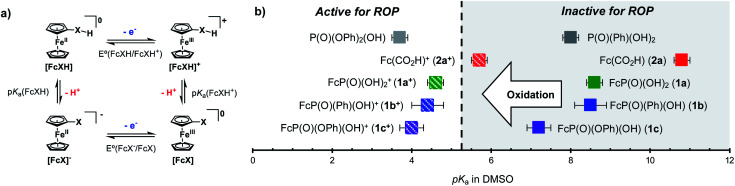
(a) Square scheme thermochemical analysis for ferrocenyl acid p*K*_a_ determination, (b) relative acidities of the neutral (solid marker) and oxidized (dashed marker) forms of the ferrocenyl acids used in this study.

Based on these experiments, we identified the redox responsive nature of our ferrocenyl acid catalysts as the key component to achieving temporal control over ROP. Thus, we first sought to understand the impact that oxidation state has on the acidity of the FcXH species. To this end, the p*K*_a_ of **1a**, **1b**, **1c**, and **2a** were determined in DMSO-d6 by titration with bases whose conjugate acids have known p*K*_a_ values in DMSO (Table S8†). The p*K*_a_s of the oxidized species **1a+**, **1b+**, **1c+**, and **2a+** were then determined through square scheme thermochemical analysis using the p*K*_a_ values of the **1a**, **1b**, **1c**, and **2a** and relevant electrochemical values [*i.e.*, *E*_1/2_(FcXH/FcXH^+^) and *E*_1/2_(FcX^−^/FcX)] (Fig. 2).^57–59^ As shown in Table S8,† the ferrocenyl carboxylic acid **2a** is the least acidic, having a p*K*_a_ of 10.8 in DMSO. The ferrocenyl phosphonic acid **1a** and the ferrocenyl (phenyl)phosphinic acid **1b** have similar p*K*_a_ values of *ca.* 8.5 in DMSO. The ferrocenyl (phenyl)phosphonic acid **1c** is the most acidic FcXH species, having a p*K*_a_ of 7.2 in DMSO. Upon oxidation, these p*K*_a_ values decrease by *ca.* 3–5 units. Although the p*K*_a_ of the ferrocenyl carboxylic acid **2a** decreases the most upon oxidation (Δp*K*_a_ = 5.1), the oxidized species (**2a+**) is the least acidic FcXH^+^ species, having a p*K*_a_ of *ca.* 6 in DMSO. The inability of **2a+** to polymerize CL suggests that the oxidized FcXH^+^ species must have a p*K*_a_ < 6 to efficiently catalyse ROP. In support of this hypothesis is the observation that phenylphosphonic acid (p*K*_a_*ca.* 8) is unable to catalyse the polymerization of CL (Table S2,† entry 1). The oxidized ferrocenyl phosphonic (**1a+**), ferrocenyl (phenyl)phosphinic (**1b+**), and ferrocenyl (phenyl)phosphonic (**1c+**) acid catalysts have similar p*K*_a_ values of ∼4 in DMSO, which is in good agreement with the p*K*_a_ value of diphenyl phosphoric acid, which is known to efficiently catalyse ROP (Fig. 2).^60,61^ These detailed measurements suggest that redox acid controlled ROP can be realized by designing acid catalysts whose p*K*_a_ values switch from greater than to less than *ca.* 5 upon application of an external stimulus.

**Fig. 3:**
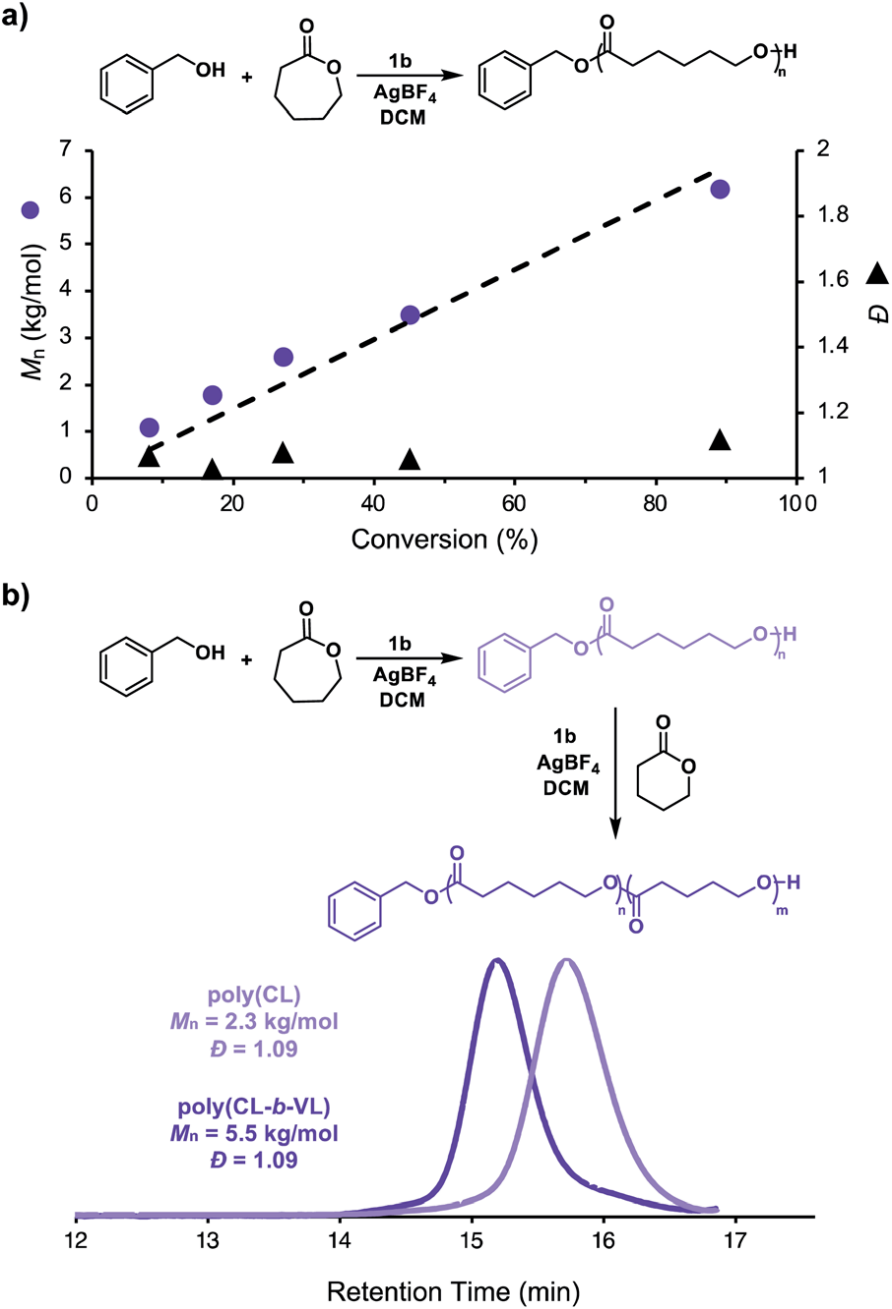
(a) Plot of *M*_*n*_*vs.* monomer conversion for the cationic ring-opening polymerization of ε-caprolactone [CL] = 4.8 M (in DCM), 1.0 mol% BnOH, 1.0 mol% [FcXH], 1.2 mol% AgBF_4_, 23 °C, (b) synthesis and GPC traces of poly(caprolactone) and poly(caprolactone-*block*-valerolactone).

We next investigated the living characteristics of redox acid controlled ROP. By varying the monomer-to-alcohol ratio, we targeted a range of *M*_*n*_s and synthesized polymers that maintained good agreement between experimental and theoretical *M*_*n*_s and low *Đ* values (Table 1, entries 10–14). Furthermore, while monitoring the polymerization of CL under optimized reaction conditions,^62^ we observed that *M*_*n*_ grows linearly with conversion (Fig. 3a). The chain-end fidelity of the resulting polyesters was assessed through the synthesis of block copolymers. Using our standard conditions, we generated a 2.3 kg mol^−1^ poly(CL) macro-initiator which was isolated and then re-subjected to identical reaction conditions to polymerize δ-valerolactone (VL). The resulting 5.5 kg mol^−1^ poly(CL-*b*-VL) diblock copolymer shows a monomodal distribution with a low *Đ* value of 1.09 (Fig. 3b). This data demonstrates the ability of our method to generate degradable polyester block polymers and shows that it is competent for monomers other than CL.

**Fig. 4:**
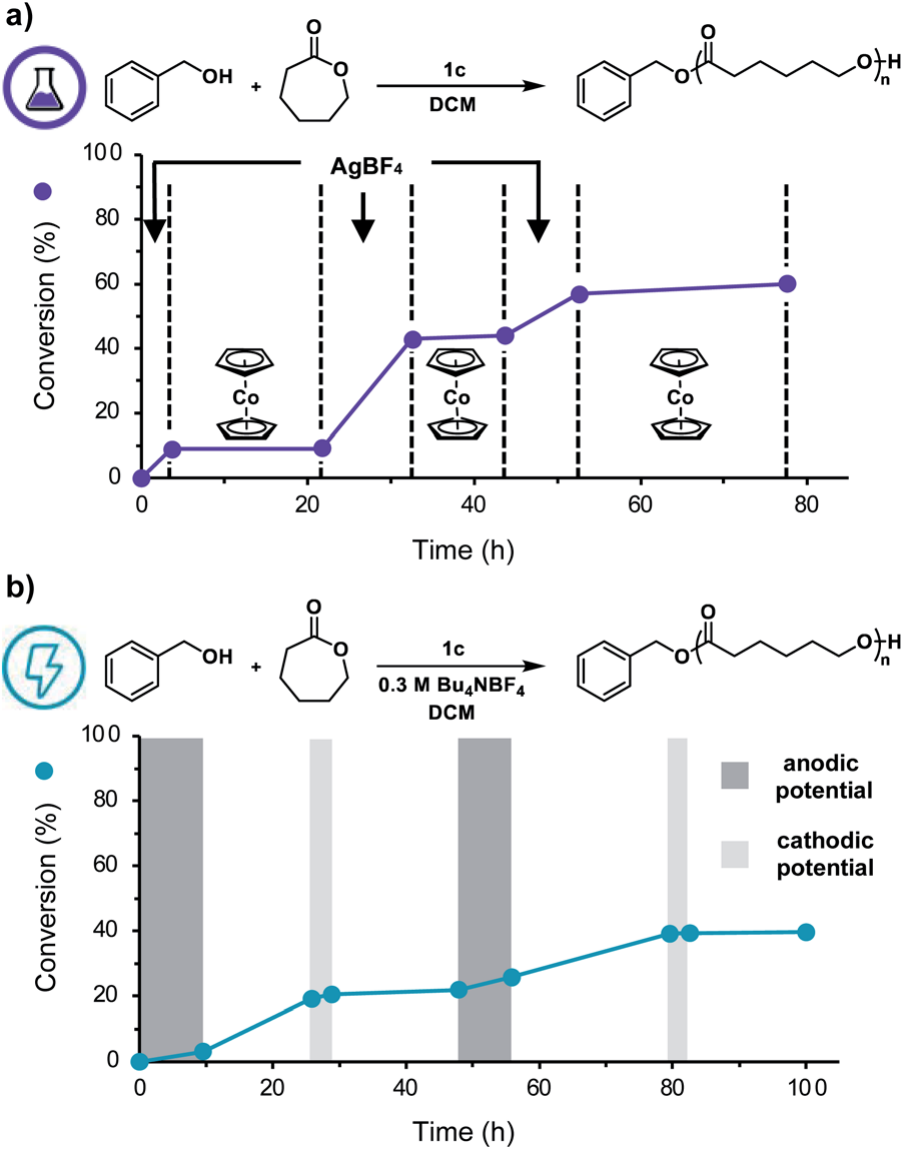
(a) Monomer conversion *vs.* time plot illustrating temporal control over ROP using AgBF_4_ to initiate polymerization and CoCp_2_ to reversibly terminate polymerization, (b) monomer conversion *vs.* time plot illustrating temporal control over ROP using anodic potential to initiate polymerization and cathodic potential to reversibly terminate polymerization.

Having shown that this system exhibits living characteristics, we next aimed to demonstrate temporal control over ROP. While addition of a chemical oxidant will oxidize the ferrocenyl acid to facilitate polymerization, we posited that subsequent addition of a chemical reductant would prompt reduction of the ferrocenyl acid to reversibly halt polymerization. Our thermochemical analysis on the acidic characteristics of the ferrocenyl acids illustrated that catalyst **1c** would be the most ideal candidate for temporally controlled polymerization as it proved to be both the most acidic and most soluble ferrocenyl acid catalyst. To test our hypothesis, we first added AgBF_4_ to a solution of CL, BnOH, and ferrocenyl acid (**1c**) in DCM. After approximately 3.5 h, cobaltocene (CoCp_2_) was added to the reaction to reversibly terminate the polymerization. This overall process was repeated two times, demonstrating excellent temporal control over the polymerization using chemical redox reagents (Fig. 4a).

We next sought to replace these chemical redox reagents with an electrochemical stimulus. Application of 2.0 mA of constant current to a solution of DCM, CL, BnOH, **1c**, and tetrabutylammonium tetrafluoroborate (Bu_4_NBF_4_) in a divided electrochemical cell fitted with reticulated vitreous carbon (RVC) electrodes, for 2.5 hours resulted in efficient polymerization (Table 1, entry 15), yielding a polymer with good agreement between experimental and theoretical *M*_*n*_s and a low *Đ* value. Importantly, no polymerization was observed in the absence of an anodic current and only uncontrolled polymerization of CL occurred in the absence of catalyst **1c** (Table 1, entries 16, 17).

To further adapt this electrochemical system for temporal control, cyclic voltammetry was performed to identify the *E*_1/2_ value of **1c** in DCM (0.78 V *vs.* Ag/AgCl, Fig. S14†). Noting this value, we applied a constant anodic potential of 1.4 V *vs.* Ag/AgCl for 9.5 h to a DCM solution of CL, BnOH, **1c**, and Bu_4_NBF_4_ in a divided electrochemical cell equipped with a platinum anode and an RVC cathode. After 16 h, a constant cathodic potential of 0.0 V *vs.* Ag/AgCl was applied for 3.0 h to reversibly terminate the polymerization. This cycling between anodic and cathodic potential was repeated once more, demonstrating excellent temporal control using an electrochemical stimulus (Fig. 4b). It should be noted that while both chemical and electrochemical methods afforded temporal control, we noticed increased tailing to lower molecular weights during electrochemical on/off experiments (Fig. S11b†). We hypothesize this observation is due to competitive oxidation of alcohol polymer chain-ends. These results illustrate the power of our redox acid platform for both efficiently controlling polymerization and enabling temporal control over polymerization—thus overcoming a challenge associated with traditional acid-mediated processes.

## Conclusions

In conclusion, we have developed a class of redox-active ferrocenyl acids that can be used to temporally control the cationic ROP of cyclic esters. The ability of these acids to reversibly promote polymerization depends upon their p*K*_a_ values in the neutral and oxidized state, suggesting that other switchable acid catalysts may be realized using similar principles. These redox acid controlled systems show excellent temporal control over the polymerization, mitigating shortcomings of traditional acid catalysed polymerizations. Furthermore, the initial chemically controlled methodology was extended to electrochemical control. When compared to other electrochemically controlled ROPs^20,63^ which utilize sensitive metal catalysts, these bench stable acid catalysts prove advantageous; however, the present system remains comparatively limited in molecular weight. We anticipate that this class of redox acid controlled polymerizations will serve as the foundation for further advancements and discovery in the field of redox acid mediated processes.

## Author contributions

M. J. S. and B. P. F. conceived this project. M. J. S. and J. H. H. conducted all polymer synthesis and optimization experiments. E. A. M. conducted all thermochemical analysis experiments. The final version of the manuscript was written with contributions from all authors.

## Conflicts of interest

There are no conflicts to declare.

## Supplementary Material

SC-012-D1SC03011F-s001
