# Supplementary material for: Reversible redox controlled acids for cationic ring-opening polymerization
Source: Chem Sci. 2021 Jul 6;12(31):10544–9. doi: 10.1039/d1sc03011f (PMC8356742; doi:10.1039/d1sc03011f)
Supplement: SC-012-D1SC03011F-s001 [file SC-012-D1SC03011F-s001.pdf]

Electronic Supplementary Material (ESI) for Chemical Science.
This journal is © The Royal Society of Chemistry 2021

Reversible Redox Controlled Acids for Cationic Ring-Opening Polymerization

Michael J. Supej, Elizabeth A. McLoughlin, Jesse H. Hsu, Brett P. Fors

Cornell University, Ithaca, New York 14853, United States

Table of Contents

General Reagent Information S2
General Analytical Information S2
General Electrochemical Cell and Electrode Set-Up S3
Synthesis of Ferrocenyl Acids S4–S6
Polymerization Procedures S7–S17
Cyclic Voltammetry Data S18–S24
Determination of pK_a Values S25–S40
NMR spectra S41-S42
References S43

General Reagent Information

ε-Caprolactone (CL) (99%, Alfa Aesar) and δ-valerolactone (VL) (≥98%, TCI) were dried over calcium hydride (CaH_2) (ACROS organics, 93% extra pure, 0–2 mm grain size) for 18 h, distilled under vacuum, and degassed by three freeze-pump-thaw cycles. Ferrocene (Fc) (98%, TCI) was used as received for synthetic procedures and purified by sublimation for electrochemical measurements. Tetrabutylammonium perchlorate (Bu_4NClO_4) (98%, TCI) and tetrabutylammonium tetrafluoroborate (Bu_4NBF_4) (98%, TCI) were purified by recrystallization from ethyl acetate three times and dried *in vacuo* at 60 °C for 18 h. 1-Ethyl-2,2,4,4,4-pentakis(dimethylamino)-2λ^5,4λ^5-catenadi (phosphazene) (Et-P_2) (≥98%, Sigma Aldrich), tert-butyl lithium (^{t}BuLi, 1.6 M in pentane, Sigma Aldrich), diethyl chlorophosphite (97%, Sigma Aldrich), phenyl dichlorophosphate (≥95%, Sigma Aldrich), phenylphosphonic dichloride (>90%, Alfa Aesar), ferrocene carboxylic acid (**2a**) (98%, Alfa Aesar), and 1,1'-ferrocenedicarboxylic acid (**2b**) (98%, Alfa Aesar) were used as received. Deuterated solvents for NMR were purchased from Cambridge Isotope Laboratories. Dichloromethane (DCM) (≥99.9%, B&J), acetonitrile (MeCN) (≥99.9%, B&J), and tetrahydrofuran (THF) (≥99.9%, Macron Fine Chemicals) were purchased from Avantor and were purified by first vigorously purging with argon for 2 h and then passing through two packed columns of neutral alumina under argon pressure. Hexanes and ethyl acetate were purchased from Fischer Scientific and used as received. Ethanol (anhydrous, 200 proof) was purchased from Koptec. Alumina (1.0, 0.3, 0.05 μm pore size) was purchased from Extec. Reticulated vitreous carbon was purchased from ERG Aerospace. Microcloth PSA (polishing paper) and Abrasive Paper (600 grit) were purchased from Buehler.

General Analytical Information

All polymer samples were analyzed using a Tosoh EcoSec HLC 8320 GPC system with two SuperHM-M columns in series at a flow rate of 0.350 mL/min. THF was used as the eluent and all number-average molecular weights (M_n), weight-average molecular weights (M_w), and dispersities ($Đ$) were determined by light scattering using a Wyatt miniDawn Treos multi-angle light scattering detector. The *dn/dc* values were calculated from light scattering in tetrahydrofuran (THF) for poly(caprolactone) [*dn/dc* = 0.068], poly(valerolactone) [*dn/dc* = 0.086], and poly(CL-*b*-VL) [*dn/dc* = 0.040] by injecting a solution of a known concentration and assuming 100% mass recovery. All reported GPC traces were acquired from refractive index chromatograms against TSKgel polystyrene standards. Nuclear magnetic resonance (NMR) spectra were recorded on a Varian 400 MHz, a Varian 600 MHz, or a Bruker 500 MHz instrument. Cyclic voltammetry experiments and electrochemical polymerizations were performed using a Bio-Logic SP-50 Potentiostat at ambient temperature under a nitrogen atmosphere. NMR samples for pK_a analysis were prepared in an inert atmosphere glovebox with freshly opened ampules of DMSO-d_6.

General Electrochemical Cell and Electrode Setup

Preparation of electrochemical cell

Divided electrochemical cells were used for all bulk electrolysis experiments. The counter (RVC), reference (Ag wire), and working (either RVC or platinum foil hung by a steel wire) electrodes were pierced through septa which were fitted to the ground glass joints (14/20). Electrical tape was used to secure the septa (Figure S1).

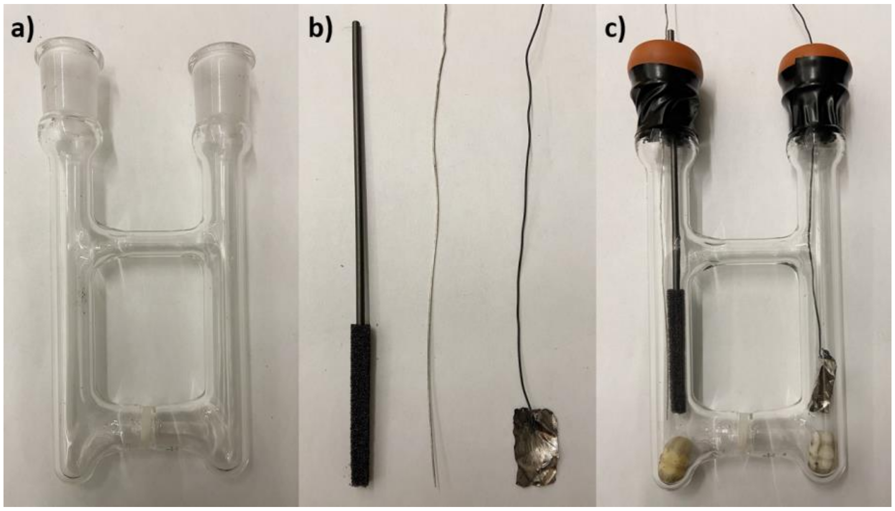

Figure S1. a) Divided electrochemical cell b) Electrodes: RVC, silver wire, and platinum foil on steel wire (left to right) c) Fully assembled electrochemical cell.

Preparation of electrodes

Reticulated vitreous carbon (RVC) electrodes were constructed by driving a 2 mm pencil lead through a 5 mm x 5 mm x 40 mm section of RVC. A platinum foil electrode was constructed by tying a steel wire (0.5 mm diameter) through a small hole at the edge of an 11mm x 18 mm x 0.1 mm piece of platinum foil. Before each experiment, the platinum foil electrode was cleaned by heating with a propane torch. Silver wire (0.5 mm diameter) functioned as the pseudo-reference electrode and was polished with fine grain (600 grit) sandpaper and rinsed with dichloromethane before each experiment. All electrodes were threaded through septa (14/20) and adjusted to maximize submerged surface area while allowing sufficient space for magnetic stirring.

Synthesis of Ferrocenyl Acids

Synthesis of diethyl ferrocenyl phosphonate

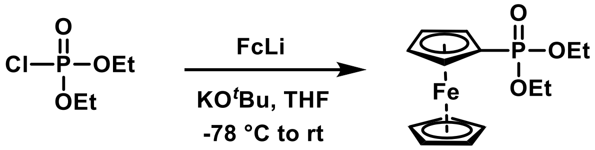

Diethyl ferrocenylphosphonate was synthesized according to a modified literature procedure.[1] In a flame-dried, 250 mL three-necked flask, ferrocene (2.5 g, 13.4 mmol, 1.4 equiv) and potassium tert-butoxide (KO^{t}Bu) (0.27 g, 2.4 mmol, 0.25 equiv) were combined. The flask was then evacuated and backfilled with N_2 3 times and left under inert atmosphere. To this flask, 50 mL of anhydrous THF were added and the reaction mixture was cooled to –78 °C, at which time the solution became heterogeneous. ^{t}BuLi solution in pentane (5.6 mL, 1.7 M, 9.52 mmol, 1.0 equiv) was added dropwise to the solution over 30 min at which point the reaction mixture turned bright red in color. The reaction was left to stir for 30 min at –78 °C, followed by the addition of diethyl chlorophosphite (2.0 mL, 13.8 mmol, 1.45 equiv) in THF (10 mL). The reaction mixture was warmed to room temperature overnight. After 16 h, the reaction mixture was slowly quenched with 100 mL of 1M NaOH, the aqueous and organic layers were separated, and the aqueous layer was extracted with DCM (3 x 30 mL). The organic layer was dried over $MgSO_4$ and evaporated to dryness *in vacuo*. The dark oil was further purified by column chromatography (SiO_2, gradient of 0 to 10% MeOH/DCM) to yield diethyl ferrocenylphosphonate as a dark, brown oil (2.23 g, 73% yield). The spectroscopic data for this compound were consistent with those reported in the literature[1]. ^{1}H NMR ($CDCl_3$, 600 MHz): δ 4.50 (m, 2 H), 4.39 (m, 2 H), 4.30 (s, 5 H), 4.12 (m, 4 H), 1.34 (t, 6 H). ^{13}C NMR ($CDCl_3$, 126 MHz): δ 71.64, 71.29, 69.95, 67.04, 61.76, 16.56. ^{31}P NMR ($CDCl_3$, 202 MHz): δ 25.80 (s).

*Synthesis of ferrocenyl phosphonic acid (**1a**)*

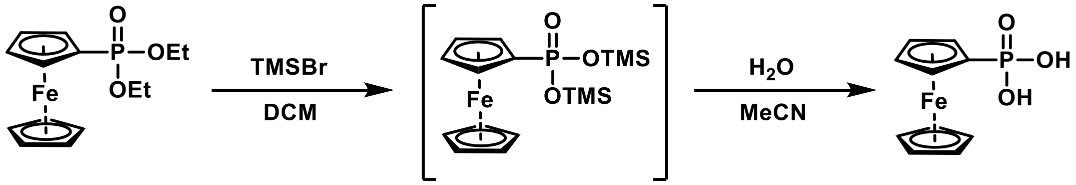

1a was synthesized according to a modified literature procedure.[1] Diethyl ferrocenylphosphonate (2.0 g, 6.2 mmol, 1 equiv) was dissolved in DCM (14 mL). To this solution, TMSBr (2.4 mL, 18.2 mmol, 2.94 equiv) was slowly added and the reaction was left to stir. After 24 h, the solvent was evaporated *in vacuo*, diluted with a 1:1 mixture of MeCN:H_2O (10 mL), and let stir for 6 h. At this time, the solution was diluted with DCM and the resulting precipitate was collected by filtration. The dark, brown solid was washed with copious amounts of H_2O then DCM, and heated to 50 °C *in vacuo* to dryness. This yielded **1a** as a hygroscopic, light-brown solid (0.765 g, 44% yield), which was stored under a blanket of N_2. The spectroscopic data for this compound were consistent with those reported in the literature.[1] ^{1}H NMR (CD_3OD, 600 MHz): δ 4.47 (m, 2 H), 4.41 (m, 2 H), 4.30 (s, 5 H). ^{13}C NMR (CD_3OD, 126 MHz): δ 135.90, 132.89, 131.79, 129.44, 72.61, 70.75. ^{31}P NMR (CD_3OD, 202 MHz): δ 23.42 (s).

*Synthesis of ferrocenyl (phenyl)phosphinic acid (**1b**)*

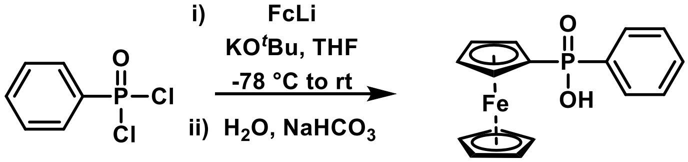

1b was synthesized according to a modified literature procedure.[1] In a flame-dried, 250 mL three-necked flask, ferrocene (2.5 g, 13.4 mmol, 1.4 equiv) and potassium tert-butoxide (KO^{t}Bu) (0.27 g, 2.4 mmol, 0.25 equiv) were combined. The flask was then evacuated and backfilled with N_2 3 times and then left under inert atmosphere. Anhydrous THF (50 mL) was added to the flask and the reaction mixture was cooled to –78 °C at which time the solution became heterogeneous. ^{t}BuLi in pentane (5.6 mL, 1.7 M, 9.52 mmol, 1.0 equiv) was added dropwise to the solution over 30 min at which point the reaction mixture turned bright red in color. The reaction was left to stir for 2 h at –78 °C, followed by the addition of phenylphosphonic dichloride (2.3 mL, 16.2 mmol, 1.71 equiv) in THF (10 mL). The reaction mixture was warmed to room temperature overnight. After 16 h, the reaction mixture was slowly quenched with 100 mL of saturated aqueous sodium bicarbonate and let stir for 3 h. The organic layer was then acidified to pH = 1 with 1M aqueous HCl. The layers were then separated, and the aqueous layer was extracted with DCM (3 x 50 mL). The organic layer was dried over Na_2SO_4 and evaporated to dryness *in vacuo*. The solid was then washed with copious amounts of ethyl acetate and dried *in vacuo* at 50 °C to yield **1b** as a light brown solid (1.79 g, 58% yield), which was stored under a blanket of N_2. The spectroscopic data for this compound were consistent with those reported in the literature.[2] ^{1}H NMR (CD_3OD, 500 MHz): δ 7.88–7.75 (m, 2 H), 7.60–7.44 (m, 3 H), 4.47 (s, 3 H), 4.24 (s, 4 H), 4.14 (s, 2 H). ^{13}C NMR (CD_3OD, 126 MHz): δ 135.90, 132.89, 131.79, 129.44, 72.61, 70.75. ^{31}P NMR (CD_3OD, 202 MHz): δ 34.12 (s).

*Synthesis of ferrocenyl (phenyl)phosphonic acid (**1c**)*

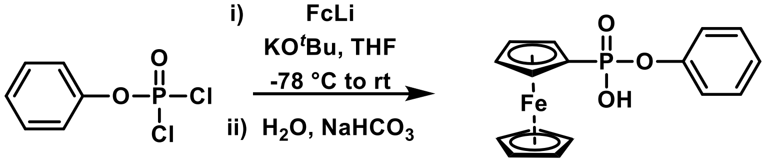

1c was synthesized according to a modified literature procedure.[1] In a flame-dried, 250 mL three-necked flask, ferrocene (2.5 g, 13.4 mmol, 1.4 equiv) and potassium tert-butoxide (KO^{t}Bu) (0.27 g, 2.4 mmol, 0.25 equiv) were combined. The flask was then evacuated and backfilled with N_2 3 times and left under inert atmosphere. Anhydrous THF (50 mL) was added to the flask and the reaction mixture was cooled to –78 °C at which time the solution became heterogeneous. ^{t}BuLi solution in pentane (5.6 mL, 1.7 M, 9.52 mmol, 1.0 equiv) was added dropwise to the solution over 30 min at which point the reaction mixture turned bright red in color. The reaction was left to stir for 2 h at –78 °C, followed by the addition of phenyl dichlorophosphate (2.5 mL, 16.5 mmol, 1.73 equiv) in THF (10 mL). The reaction mixture was warmed to room temperature overnight. After 16 h, the reaction mixture was slowly quenched with 100 mL of saturated aqueous sodium bicarbonate and stirred for 3 h. The organic layer was then acidified to pH = 1 with 1M aqueous HCl. The organic and aqueous layers were separated, and the aqueous layer was extracted with DCM (3 x 30 mL). The organic layer was dried over Na_2SO_4 and evaporated to dryness *in vacuo*. The solid was washed with copious amounts of ethyl acetate and dried *in vacuo* at 50 °C to yield **1c** as a light brown solid (1.29 g, 40% yield), which was stored under a

blanket of N_2. ^1H NMR (CD_3OD, 500 MHz): δ 7.34–7.28 (m, 2 H), 7.18–7.09 (m, 3 H), 4.55–4.45 (m, 4 H), 4.33 (s, 5 H). ^{13}C NMR (CD_3OD, 126 MHz): δ 150.95, 129.15, 124.32, 120.54, 71.37, 69.59. ^{31}P NMR (CD_3OD, 202 MHz): δ 22.55 (s). HRMS (ESI-MS): calculated for $C_{16}H_{14}FePO_3^-$ [M-H$^+$] 341.00355, found 341.00308.

Synthesis of phenyl phosphonic acid

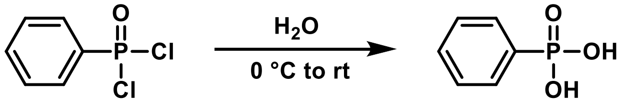

Phenylphosphonic dichloride (0.71 mL, 5 mmol) was added to a glass reaction tube and cooled to 0 °C before water (3.0 mL, 166 mmol) was slowly added to the reaction tube. The cloudy solution was warmed to room temperature and stirred for 8 h. The reaction mixture was washed with 2 mL of 1 M HCl, extracted with ethyl acetate (3 x 5 mL), dried over Na_2SO_4, and evaporated to dryness *in vacuo*. The resulting wet powder was then further dried by azeotroping with benzene yielding phenyl phosphonic acid as a white powder (0.466 g, 59.0% yield), which was stored under a blanket of N_2. The spectroscopic data for this compound were consistent with those reported for the commercial compound sold by Sigma Aldrich. ^1H NMR (CD_3OD, 500 MHz): δ 7.78–7.46 (m, 2 H), 7.44–7.18 (m, 3 H). ^{13}C NMR (CD_3OD, 126 MHz): δ 133.89, 132.79, 131.88, 129.37. ^{31}P NMR (CD_3OD, 202 MHz): δ 16.18 (s).

Polymerization Procedures

General Procedure for Solvent-Free Redox Acid Controlled Cationic Ring Opening Polymerization of ε-Caprolactone

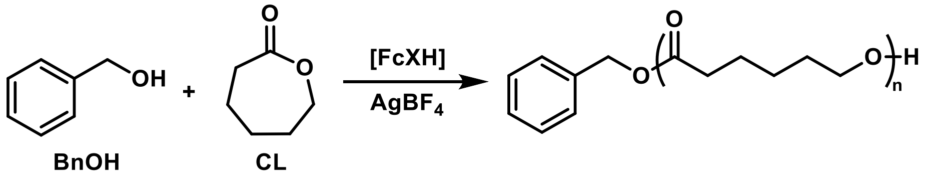

$AgBF_4$ (12.0 mg, 0.062 mmol, 1.2 equiv) and the ferrocene acid catalyst (0.05 mmol, 1.0 equiv) were added to an oven-dried one-dram vial equipped with a stir bar. The vial was then evacuated and backfilled with N_2 and charged with benzyl alcohol (5.2 μL, 0.05 mmol, 1.0 equiv) followed by ε-caprolactone (0.55 mL, 5 mmol, 100 equiv). The reaction was stirred at room temperature for the indicated time, and then quenched by addition of excess sodium dithionite and diluted with 0.4 mL of THF for analysis by ^1H NMR and GPC.

Table S1. FcXH mediated polymerization of ε-caprolactone in the absence of $AgBF_4$.

**Entry**^{a}	**Catalyst**	**Conversion**	$M_{n,Exp}$ **(kg/mol)**	*Đ*
1	1a	<5%	—	—
2	1b	<5%	—	—
3	1c	<5%	—	—
4	2a	<5%	—	—
5	2b	<5%	—	—

^{a}Reaction Conditions: [CL] = 9.1 M (neat), 1.0 mol % [FcXH], [CL]:[BnOH]:[FcXH] = 100:1.0:1.0.

General Procedure for Redox Acid Controlled Cationic Ring Opening Polymerization of ε-Caprolactone with Catalyst ***1b*** *in dichloromethane*

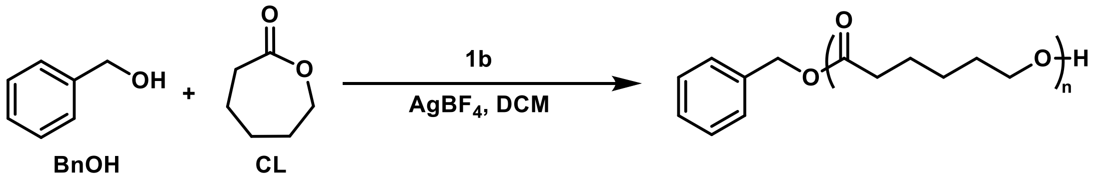

$AgBF_4$ (12.0 mg, 0.062 mmol, 1.2 equiv) and catalyst **1b** (16.0 mg, 0.05 mmol, 1.0 equiv) were added to an oven-dried one-dram vial equipped with a stir bar. The vial was evacuated and backfilled with N_2 and sequentially charged with benzyl alcohol (5.2 μL, 0.05 mmol, 1.0 equiv), DCM (0.5 mL), and ε-caprolactone (0.55 mL, 5.0 mmol, 100 equiv). The reaction was stirred at room temperature for the indicated time, and then quenched by addition of excess sodium dithionite and diluted with 0.4 mL of THF for analysis by ^1H NMR and GPC.

Procedure for the Control Reaction of ε-Caprolactone in the Presence of Phenylphosphonic Acid

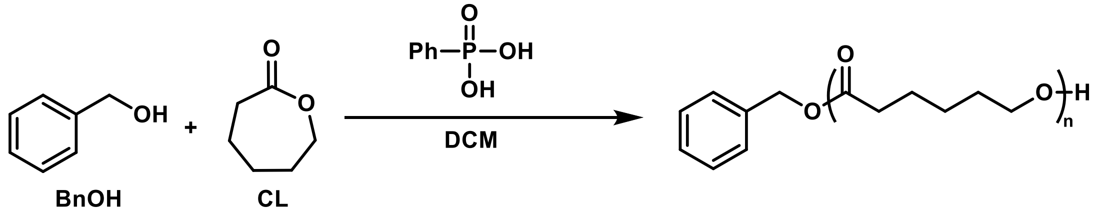

Phenylphosphonic acid (7.9 mg, 0.05 mmol, 1.0 equiv) was added to an oven-dried one-dram vial equipped with a stir bar. The vial was evacuated and backfilled with N_2 and sequentially charged with benzyl alcohol (5.2 µL, 0.05 mmol, 1.0 equiv), DCM (0.5 mL), and ε-caprolactone (0.55 mL, 5.0 mmol, 100 equiv). The reaction was stirred at room temperature for 18 h, and then quenched by addition of excess sodium dithionite and diluted with 0.4 mL of THF for analysis by ^{1}H NMR and GPC.

Table S2. Polymerization of ε-caprolactone in the presence of phenylphosphonic acid.

Entry[a]	Catalyst	Conversion	$M_{n,Exp}$ (kg/mol)	$Đ$
1	$[P(O)(Ph)(OH)_2]$	0%	—	—

[a]Reaction Conditions: [CL] = 4.8 M, $[CL]:[BnOH]:[P(O)(Ph)(OH)_2]$ = 100:1.0:1.0.

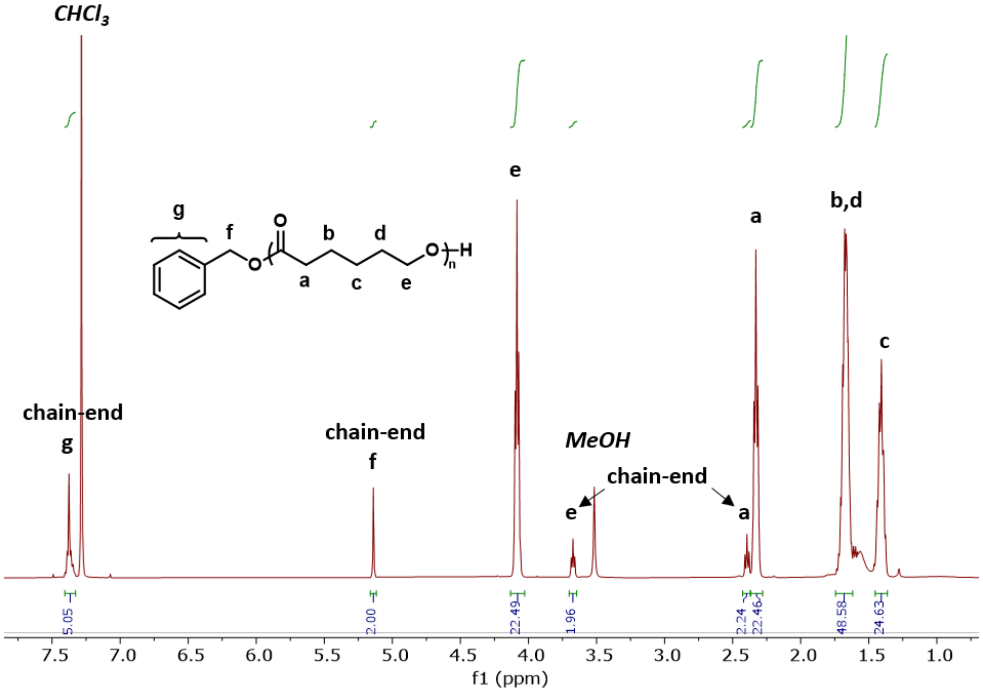

Figure S2. Representative ^{1}H NMR spectrum of poly(ε-caprolactone) acquired in $CDCl_3$ showing the benzyl group chain-end (Table 1, entry 13).

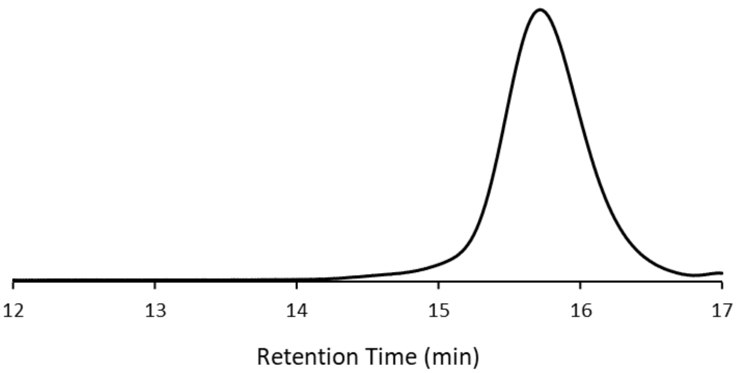

Figure S3. Representative GPC trace of poly(ε-caprolactone) (Table 1, entry 13).

*Procedure for Redox Acid Controlled Cationic Ring Opening Polymerization with Catalyst **1b** while Metering in Caprolactone Over Time (Table 1, entry 10)*

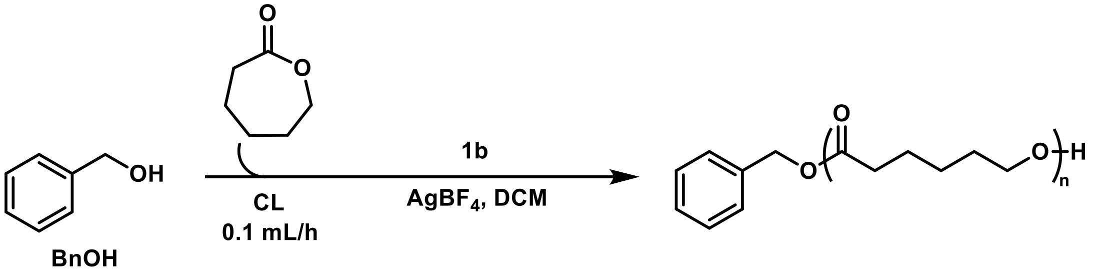

$AgBF_4$ (12.0 mg, 0.062 mmol, 1.2 equiv) and catalyst **1b** (16.0 mg, 0.05 mmol, 1.0 equiv) were added to an oven-dried one-dram vial equipped with a stir bar. The vial was evacuated and backfilled with N_2 and sequentially charged with benzyl alcohol (2.6 μL, 0.025 mmol, 0.5 equiv) and DCM (0.1 mL). A solution of caprolactone (0.55 mL, 5.0 mmol, 100 equiv) in DCM (0.4 mL) was metered into the reaction at a rate of 0.1 mL/h using a syringe pump. Following the addition, the reactions was stirred at room temperature for 18 h and then terminated by addition of excess sodium dithionite and diluted with 0.4 mL of THF for analysis by ^{1}H NMR and GPC. ($M_{n,Exp}$ = 10.9 kg/mol, $M_{n,Theo}$ = 11.3 kg/mol, $Đ$ = 1.26).

Note: As we aimed for higher molecular weight polymers (M_n > 8 kg/mol), we noticed a greater divergence between experimental and theoretical molecular weights. We believe this was due to the simultaneous occurrence of the Activated Monomer Mechanism (AMM) and Activated Chain-End Mechanism (ACEM) at lower concentrations of initiator. Based on work done by Navarro and coworkers[3] on the acid catalyzed polymerization of cyclic carbonates, we metered in monomer over time during the above polymerization (leading to a lower monomer concentration and lower [M]:[I] ratio at early reaction timepoints) to favor the AMM and achieve better agreement between experimental and theoretical molecular weights. This strategy was successful in generating a 10.9 kg/mol polymer of poly(ε-caprolactone), suggesting that this approach may be generalized for other higher molecular weight polymers.

*Procedure for Redox Acid Controlled Cationic Ring Opening Polymerization of δ-Valerolactone with Catalyst **1b***

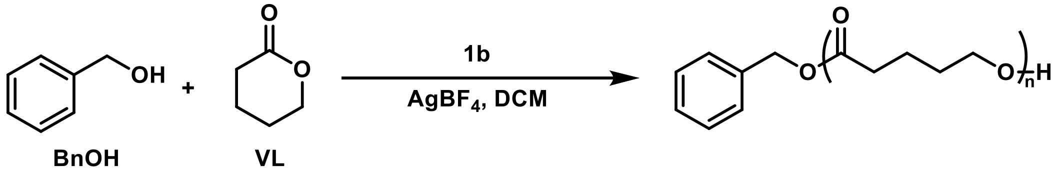

$AgBF_4$ (12.0 mg, 0.062 mmol, 0.6 equiv) and catalyst **1b** (16.0 mg, 0.05 mmol, 0.5 equiv) were added to an oven-dried one-dram vial equipped with a stir bar. The vial was evacuated and backfilled with N_2 and sequentially charged with benzyl alcohol (10.0 µL, 0.096 mmol, 1.0 equiv), DCM (0.63 mL), and δ-valerolactone (0.46 mL, 5.0 mmol, 52 equiv). After stirring at room temperature for 18 h, the reaction was terminated by addition of excess sodium dithionite and diluted with 0.4 mL of THF for analysis by ^{1}H NMR and GPC.

Table S3. Polymerization of δ- valerolactone with catalyst **1b**.

Entry[a]	Catalyst	[VL]:[BnOH]:[1b]:[$AgBF_4$]	Conversion	$M_{n,Exp}$ (kg/mol)	$M_{n,Theo}$ (kg/ mol)	Đ
1	1b	52:1.0:0.5:0.6	95%	4.4	4.9	1.12
2	1b	26:1.0:0.5:0.6	88%	2.2	2.4	1.22
3	1b	10:1.0:0.5:0.6	95%	1.3	1.1	1.23

[a]Reaction Conditions: [VL] = 4.6 M, 1.0 mol % [FcXH], 1.2 mol % $AgBF_4$. [b]$M_{n,Theo}$ = [VL]/[BnOH] X MW_{CL} X Conversion + $MW_{BnOH.}$

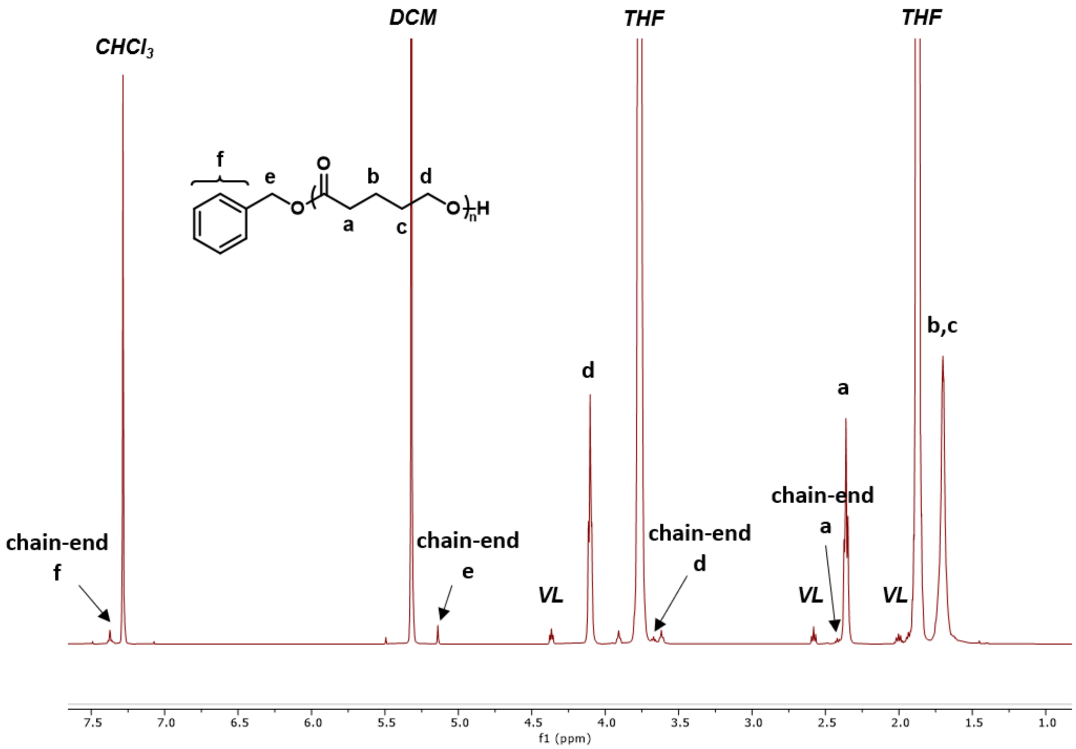

Figure S4. Representative ^{1}H NMR spectrum of poly(*δ*-valerolactone) acquired in $CDCl_3$ showing the benzyl group chain-end (Table S2, entry 1).

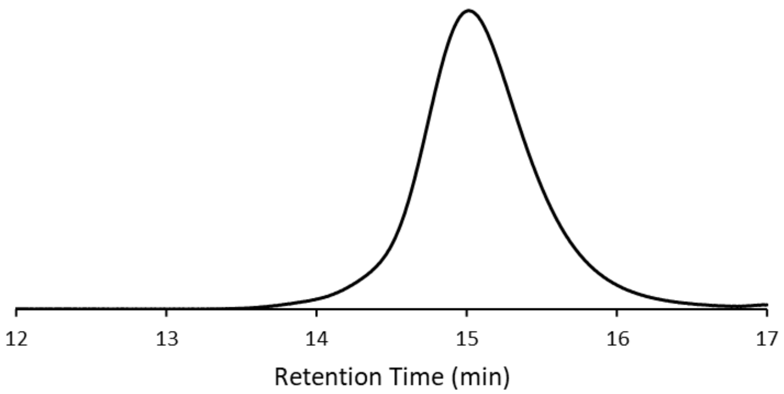

Figure S5. Representative GPC trace of poly(*δ*-valerolactone) (Table S2, entry 1).

General Procedure for Redox Acid Controlled Cationic Ring Opening Polymerization to Generate Poly(CL-b-VL) Block Copolymer

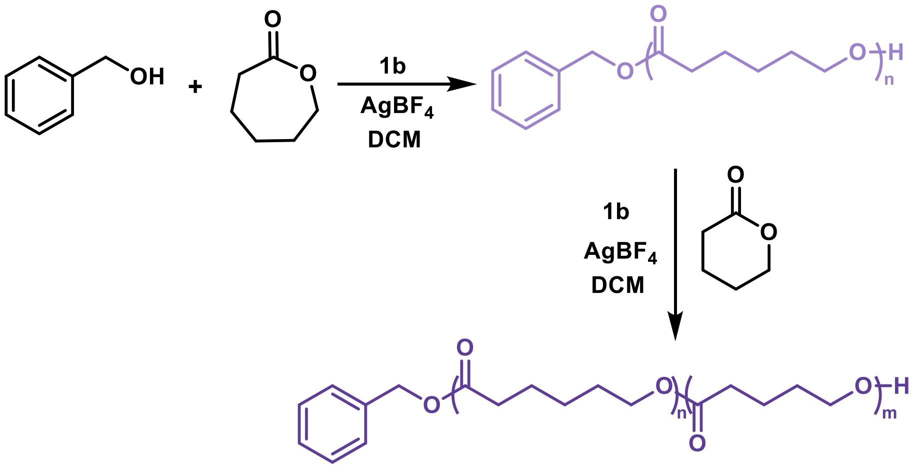

$AgBF_4$ (12.0 mg, 0.062 mmol, 1.2 equiv) and catalyst **1b** (16.0 mg, 0.05 mmol, 1.0 equiv) were added to an oven-dried one-dram vial equipped with a stir bar. The vial was evacuated and backfilled with N_2 and sequentially charged with benzyl alcohol (20 µL, 0.19 mmol, 4.0 equiv), DCM (0.48 mL), and ε-caprolactone (0.55 mL, 5.0 mmol, 100 equiv). After 18 h, the reaction was terminated by addition of excess sodium dithionite and diluted with 0.4 mL of THF. Poly(CL) was then precipitated from methanol at 0 °C, dried *in vacuo*, and then analyzed by ^1H NMR and GPC. To achieve chain extension with δ-valerolactone, $AgBF_4$ (1.2 mg, 6.2 µmol, 0.19 equiv), catalyst **1b** (1.6 mg, 5.0 µmol, 0.16 equiv), and the previously prepared poly(CL) macroinitiator (71.0 mg, 0.031 mmol, 1.0 equiv) were added to an oven-dried one-dram vial that was equipped with a magnetic stir bar. The vial evacuated and backfilled with N_2 and sequentially charged with DCM (0.21 mL) and δ-valerolactone (90 µL, 0.98 mmol, 32 equiv). After stirring at room temperature for 2 h, the reaction was terminated by addition of excess sodium dithionite, diluted with 0.4 mL of THF, and analyzed by ^1H NMR and GPC.

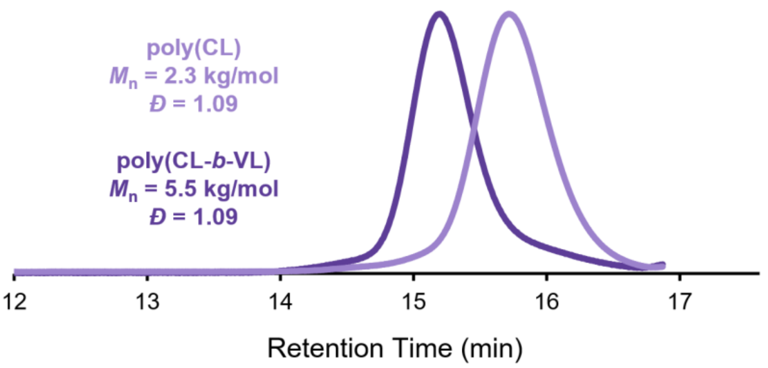

Figure S6. GPC traces of poly(CL) and poly(CL-*b*-VL) diblock copolymer ($M_{n,Theo}$ = 4.2 kg/mol, $M_{n,Exp}$ = 5.5 kg/mol, $Đ$ = 1.09).

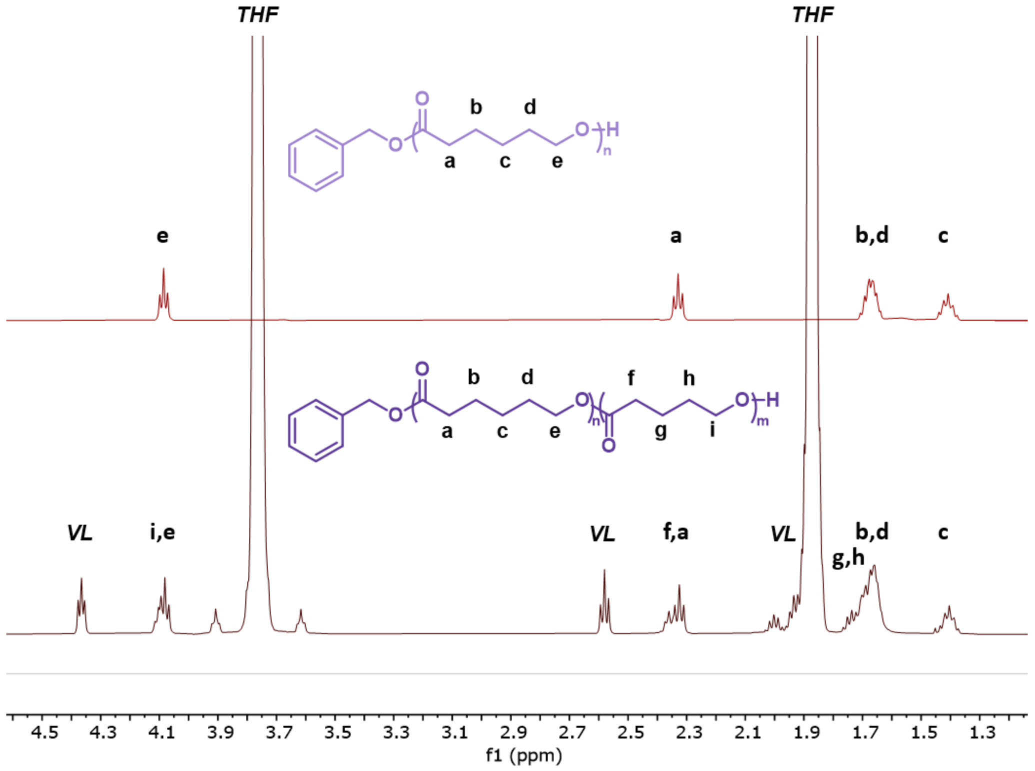

Figure S7. ^{1}H NMR spectra of poly(CL) macroinitiator (top) and poly(CL-*b*-VL) diblock copolymer (bottom) in $CDCl_3$.

*General Procedure for Chemically Initiating and Terminating Redox Acid Controlled Cationic Ring Opening Polymerization of Caprolactone with Catalyst **1c***

In a nitrogen-filled glovebox, $AgBF_4$ (12.0 mg, 0.062 mmol, 1.2 equiv) and catalyst **1c** (17.1 mg, 0.05 mmol, 1.0 equiv) were added to a one-dram vial equipped with a magnetic stir bar. The vial was sequentially charged with benzyl alcohol (5.2 μL, 0.05 mmol, 1.0 equiv), DCM (0.75 mL), and ε-caprolactone (0.55 mL, 5.0 mmol, 100 equiv). After an "on" period of 3 h 36 min at room temperature, an aliquot (0.05 mL) was taken and cobaltocene (35.0 mg, 0.185 mmol, 3.7 equiv) was subsequently added. This procedure was cycled twice more where aliquots preceded the addition of $AgBF_4$ (46.7 mg, 0.240 mmol, 4.8 equiv) after an 18 h 7 min "off" period, cobaltocene (55.8 mg, 0.295 mmol, 5.9 equiv) after a 10 h 46 min "on" period, $AgBF_4$ (70.0 mg, 0.362 mmol, 7.0 equiv) after an 11 h 14 min "off" period, and cobaltocene (76.6 mg, 0.405 mmol, 8.1 equiv) after an 8 h 48 min "on" period. A final aliquot was taken after a 25 h 10 min "off" period. All aliquots were quenched by excess sodium dithionite outside of the glovebox and analyzed by ^1H NMR and GPC.

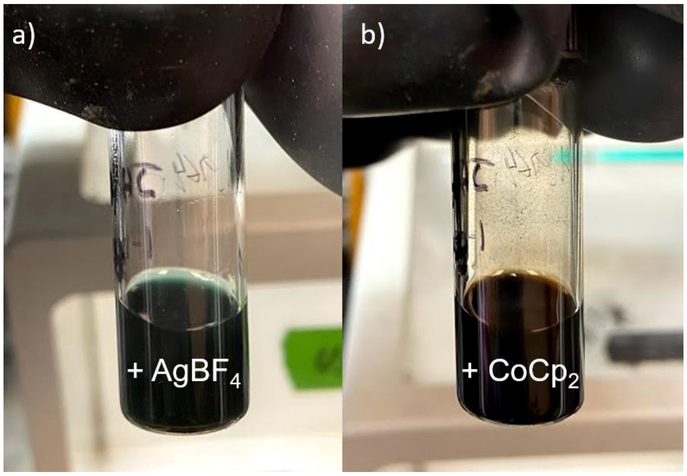

Figure S8. a) Chemically controlled ROP after the addition of $AgBF_4$ and b) after subsequent addition of $CoCp_2$.

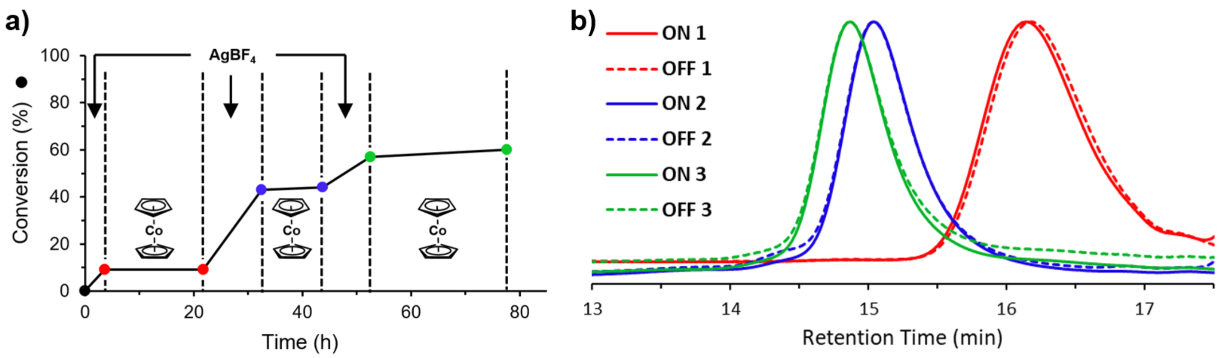

Figure S9. a) Monomer conversion vs. time plot illustrating temporal control over ROP using $AgBF_4$ to activate for polymerization and $CoCp_2$ to reversibly terminate polymerization b) Representative GPC traces for on/off experiment.

Table S4. Chemically Initiated/Terminated Cationic Ring Opening Polymerization of Caprolactone with Catalyst **1c**.

Entry	Time (h)	Conversion (%)	$M_{n,Theo}$ (kg/ mol)	$M_{n,Exp}$ (LS) (kg/mol)	$Đ$ (LS)	$M_{n,Exp}$ (RI) (kg/mol)	$Đ$ (RI)
1	3.6	9.1	1.0	1.4	1.09	1.4	1.19
2	21.7	9.1	1.0	1.1	1.10	1.4	1.18
3	32.5	43	4.9	7.3	1.37	5.1	1.18
4	43.7	44	5.0	5.4	1.23	5.2	1.18
5	52.5	57	6.5	7.7	1.50	6.2	1.16
6	77.7	60	6.8	5.7	1.50	6.1	1.16

Note: A significant background signal was observed in light scattering (LS) data after the repeated addition of oxidant/reductant and accumulation of excess $AgBF_4$/$CoCp_2$ (entries 3-6). Due to this inserted error, additional M_n and $Đ$ values were acquired and are reported by refractive index (RI) detection calibrated against TSKgel polystyrene standards. It should be noted that despite this effect on LS data, no effect on polymerization efficiency was observed.

*General Procedure for Electrochemical Redox Acid Controlled Cationic Ring Opening Polymerization of Caprolactone with Catalyst **1c** (Constant Current)*

To an oven dried electrochemical cell, a magnetic stir bar was added to both the working and counter compartment. The cell was equipped with a RVC anode and cathode and then sealed with rubber septa. The electrochemical cell was then evacuated and backfilled with nitrogen. Once the electrochemical cell had cooled to room temperature, **1c** (100 mg, 0.29 mmol, 0.5 equiv) was added to the working compartment and Bu_4NBF_4 (400 mg, 1.2 mmol) was added to both the working and counter compartments. The cell was then evacuated and backfilled with positive pressure of nitrogen three times. Then the working compartment was charged with CL (3.30 mL, 29.8 mmol, 51 equiv), benzyl alcohol (60 μL, 0.58 mmol, 1 equiv), and DCM to bring the total volume to 6.0 mL. The counter compartment was charged with 6.0 mL of DCM. The leads of a DC power supply were connected to the electrodes. Stirring began and an anodic current (2.0 mA) was applied for a desired amount of time, and the reaction was left to stir at room temperature for 18 h. Aliquots were taken by syringe under a blanket of N_2 and were quenched with excess sodium dithionite in THF and then analyzed by ^{1}H NMR and GPC.

Table S5. Control Reactions for the Electrochemical Redox Acid Controlled Polymerization of Caprolactone (Entry 15-17 in Table 1).

Entry[a]	Applied Current	Catalyst	Conversion	$M_{n,Exp}$ (kg/mol)	$M_{n,Theo}$ (kg/mol)	$Đ$
1	2.0 mA	1c	65%	2.8	3.8	1.08
2	2.0 mA	None	>99%	8.8	5.9	1.86
3	None	1c	0%	—	—	—

[a]Reaction Conditions: [CL] = 4.98 M, [CL]:[BnOH]:[FcXH] = 51:1.0:0.5, 2.0 mA constant current, RVC anode, RVC cathode.

*General Procedure for Electrochemically Initiating and Terminating Redox Acid Controlled Cationic Ring Opening Polymerization of Caprolactone with Catalyst **1c***

To an oven dried electrochemical cell, a magnetic stir bar was added to both the working and counter compartments. In a nitrogen-filled glovebox, **1c** (27.5 mg, 0.0804 mmol, 1.1 equiv) was added to the working compartment, and Bu_4NBF_4 (400 mg, 1.21 mmol) was added to both the working and counter compartments. Then benzyl alcohol (7.86 μL, 0.0756 mmol, 1 equiv) was added to the working compartment and CL (0.836 mL, 7.54 mmol, 100 equiv) was added to the working and counter compartments. DCM was added to both compartments to bring the total volume of each side to 4.0 mL. The working compartment was equipped with a platinum foil anode, which was hung by a steel wire. The counter compartment was fitted with an RVC cathode, and a silver wire pseudo-reference electrode. The electrochemical cell was sealed with rubber septa and electrical tape before pumped out of the glovebox. The silver wire pseudo-reference was referenced to a Ag/AgCl reference electrode for all reported applied potential values. Applied voltages were chosen to maximize the delivered current while minimizing unintended redox events (see Figure S11). Potentiostat leads were connected to the electrochemical cell, and the polymerization was initiated by first applying 1.4 V (vs. Ag/AgCl) of anodic potential for 9 h 30 min. Near completion of this oxidation was signified by a dramatic color change from yellow to dark blue (Figure S10) as well as a large reduction in current (Figure S12). After allowing the reaction to stir at room temperature for an additional 16 h 20 min, 0.0 V (vs. Ag/AgCl) of cathodic potential was applied to the cell for an additional 3 h to reversibly terminate the reaction. The end point of this reduction was indicated by a color change back to the original yellow color (Figure S10) as well as an inflection point in the corresponding current vs. time plot (Figure S13). The reaction was then left off for 19 h before being reinitiated by 1.3 V (vs. Ag/AgCl) of anodic potential for 8 h. The reaction was allowed to stir for 23 h 40 min before being reversibly terminated by application of 0.0 V (vs. Ag/AgCl) of cathodic potential for 3 h. The reaction was then left off for 17 hours 30 min. Aliquots were taken after each pulse and stirring period and were quenched by excess sodium dithionite before analysis by ^{1}H NMR and THF GPC.

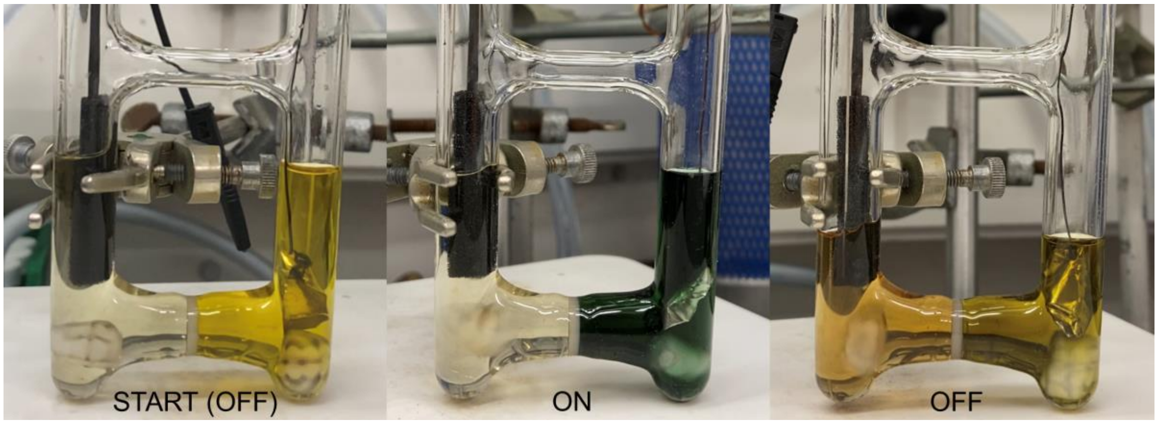

Figure S10. Electrochemically controlled ROP after the application of anodic potential (ON) and after subsequent application of cathodic potential (OFF).

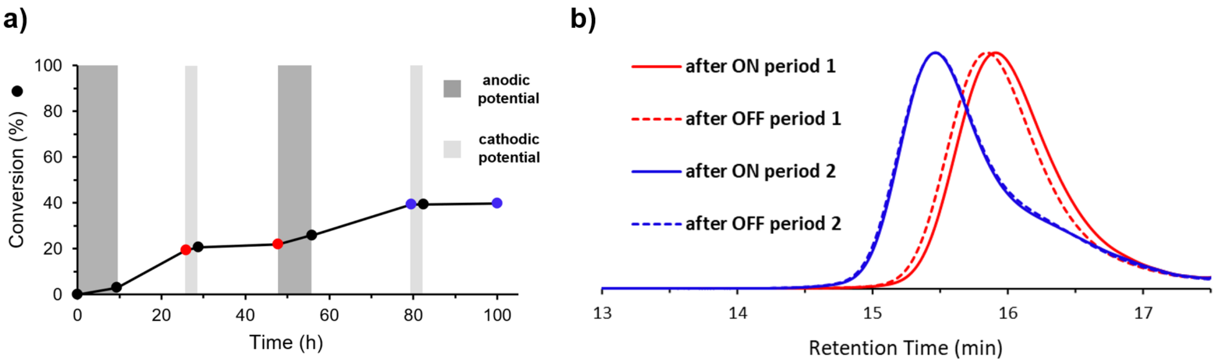

Figure S11. a) Monomer conversion vs. time plot illustrating temporal control over ROP using anodic potential to initiate polymerization and cathodic potential to reversibly terminate polymerization b) Representative GPC traces for on/off experiment. We attribute the tailing toward low molecular weight to competitive oxidation of the alcohol chain ends, which are capable of being oxidized (E_{ox} = 0.5–1.3 V vs. SCE)[4] under our electrochemical conditions.

Table S6. Electrochemically Initiated/Terminated Cationic Ring Opening Polymerization of Caprolactone with Catalyst **1c**.

Entry	Time (h)	Conversion (%)	$M_{n,Theo}$ (kg/mol)	$M_{n,Exp}$ (kg/mol)	*Đ*
1	25.8	19	2.2	1.9	1.03
2	47.8	22	2.5	2.0	1.03
3	79.5	39	4.5	2.8	1.09
4	100	40	4.6	2.5	1.14

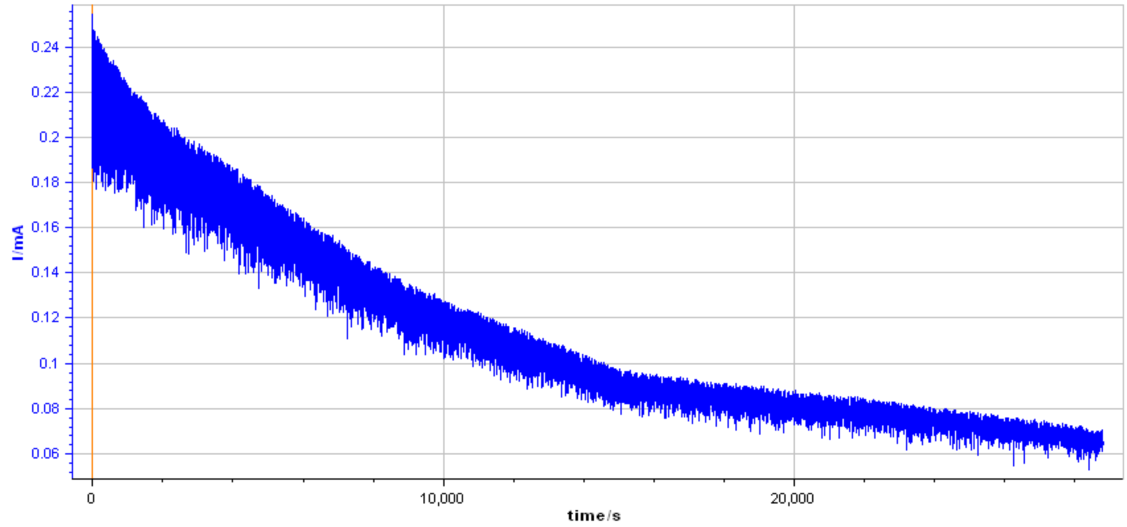

Figure S12. Representative current vs. time plot corresponding to an applied anodic potential of 1.4 V (vs. Ag/AgCl).

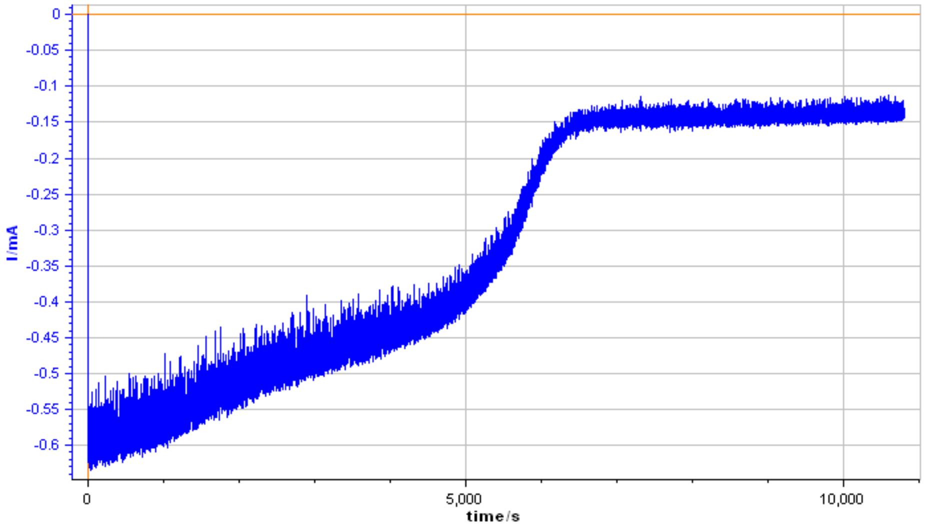

Figure S13. Representative current vs. time plot corresponding to an applied cathodic potential of 0.0 V (vs. Ag/AgCl).

Cyclic Voltammetry Data

General Procedure

Cyclic voltammetry experiments were performed using a Bio-Logic SP-50 Potentiostat at ambient temperature under a nitrogen atmosphere. A typical electrochemical cell consisted of a three-electrode setup using a glassy carbon working electrode, a platinum wire counter electrode, and a Ag/AgCl reference electrode. All electrochemical experiments in DMSO were performed with 0.1 M tetrabutylammonium perchlorate (Bu_4NClO_4) as supporting electrolyte. The glassy carbon working electrode was polished between each scan. All potentials recorded in DMSO are referenced to the $Fc^{0/+}$ couple (0.0 V).

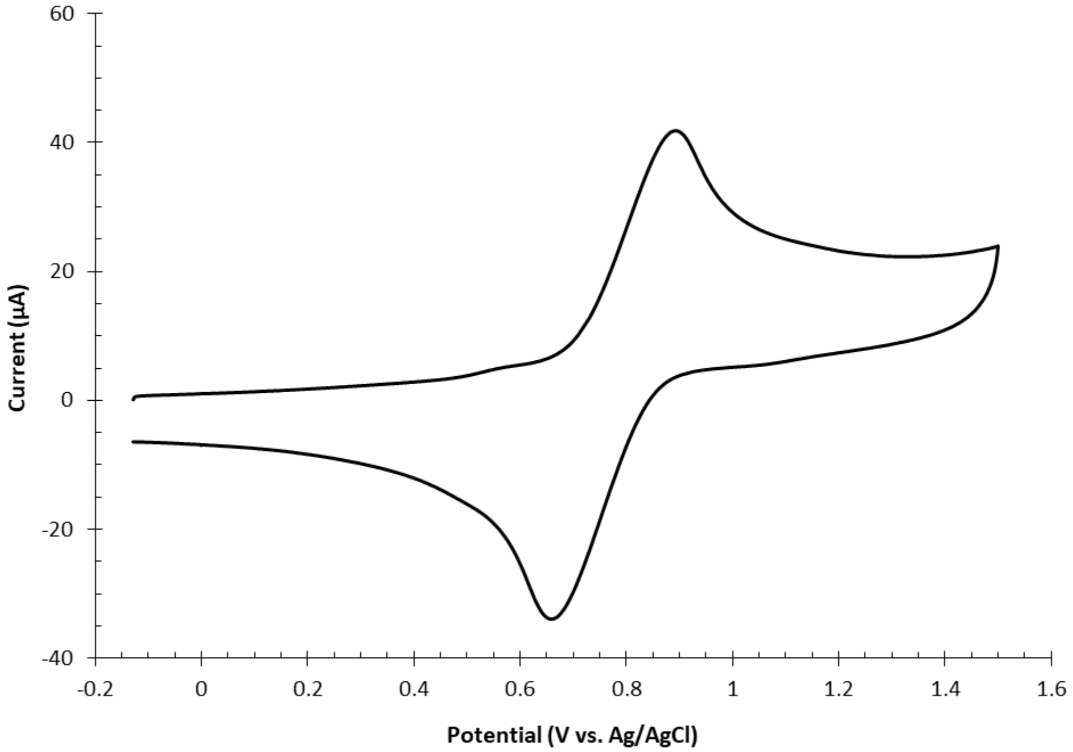

Figure S14. Cyclic voltammogram of **1c** (1.0 mM) in 0.1 M Bu_4NBF_4 in DCM. Scan rate 500 mV/s. Anodic sweep.

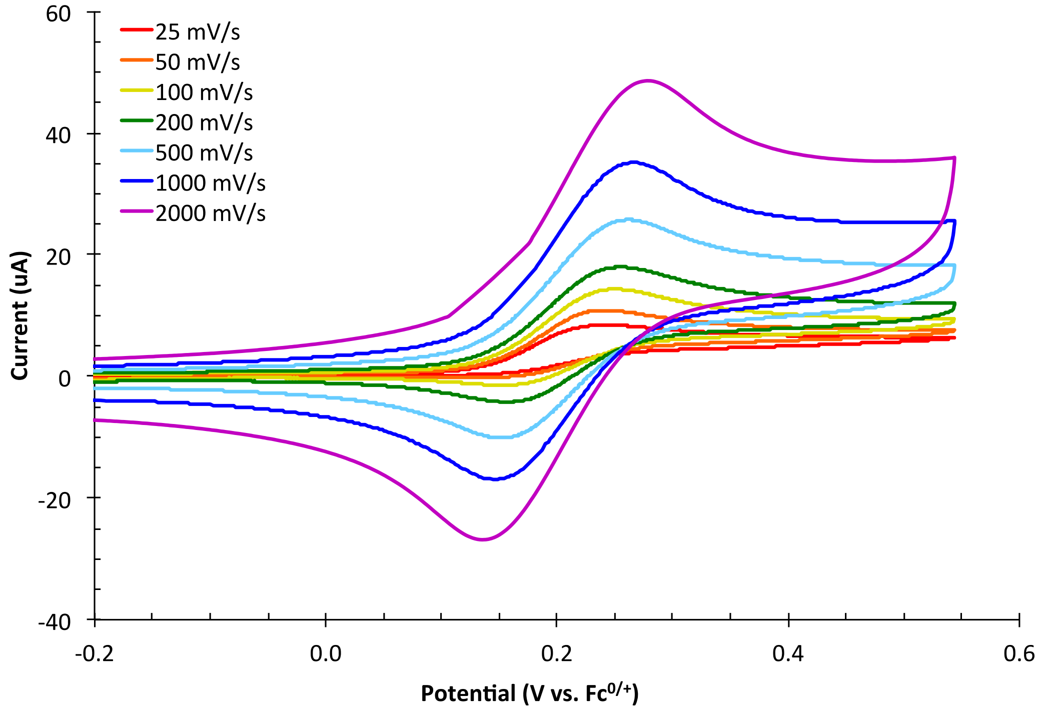

Figure S15. Cyclic voltammograms of **2a** (1.0 mM) in 0.1 M Bu_4NClO_4 in DMSO at various scan rates. Anodic sweep.

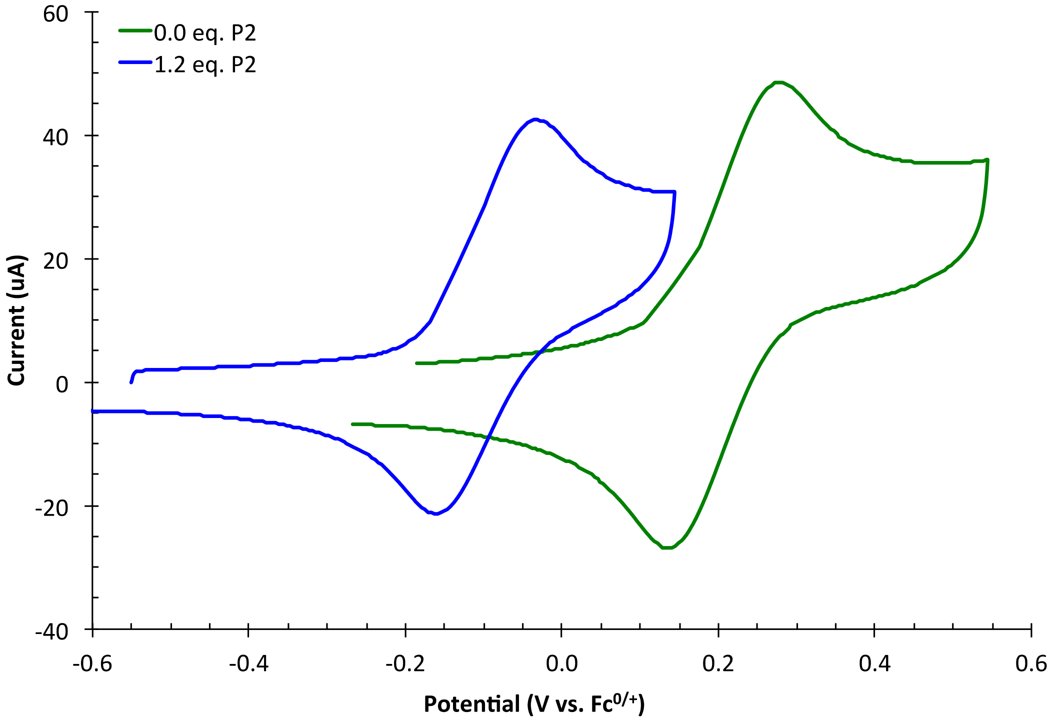

Figure S16. Cyclic voltammograms of 2a (1.0 mM) in the absence (green trace) and presence of Et-P_2 (1.2 mM, blue trace) in 0.1 M Bu_4NClO_4 in DMSO. Scan rate 2000 mV/s.

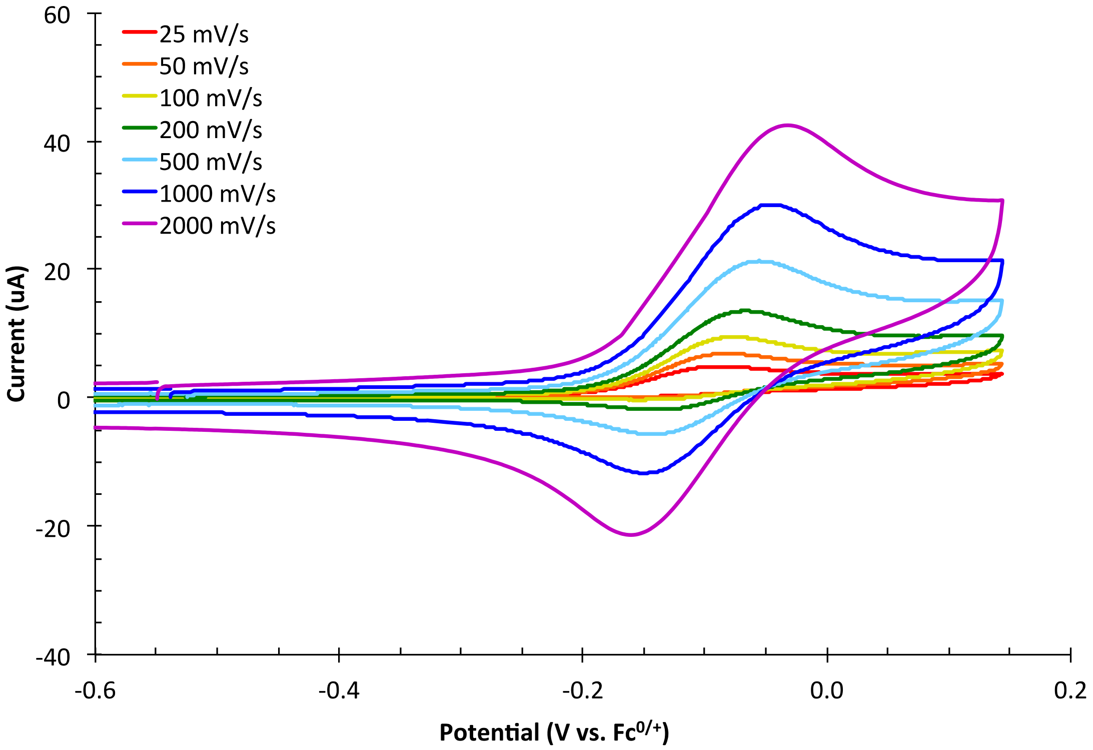

Figure S17. Cyclic voltammograms of **2a** (1.0 mM) in the presence of Et-P_2 (1.2 mM) in 0.1 M Bu_4NClO_4 in DMSO at various scan rates. Anodic sweep.

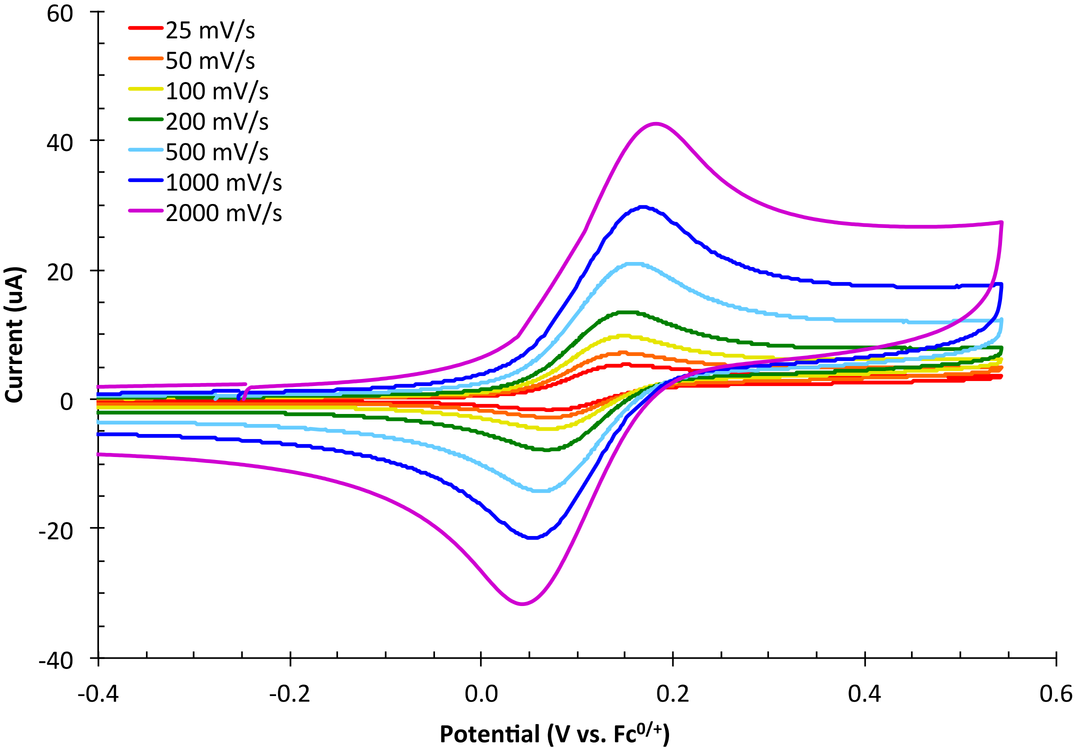

Figure S18. Cyclic voltammograms of 1a (1.0 mM) in 0.1 M Bu_4NClO_4 in DMSO at various scan rates. Anodic sweep.

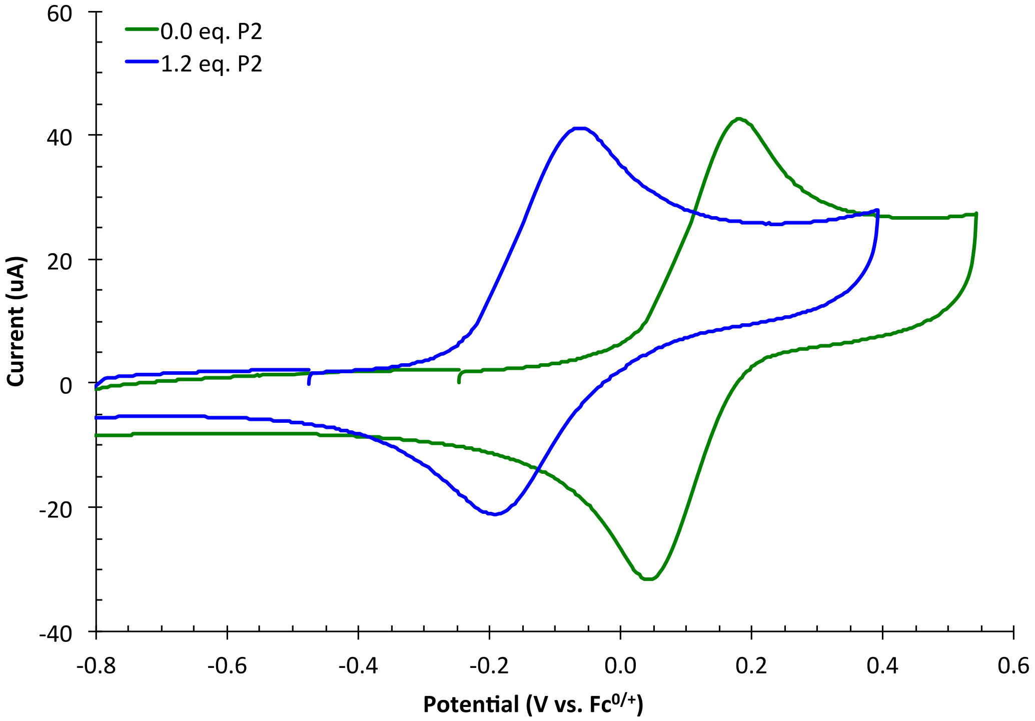

Figure S19. Cyclic voltammograms of **1a** (1.0 mM) in the absence (green trace) and presence of Et-P_2 (1.2 mM, blue trace) in 0.1 M Bu_4NClO_4 in DMSO. Scan rate 2000 mV/s.

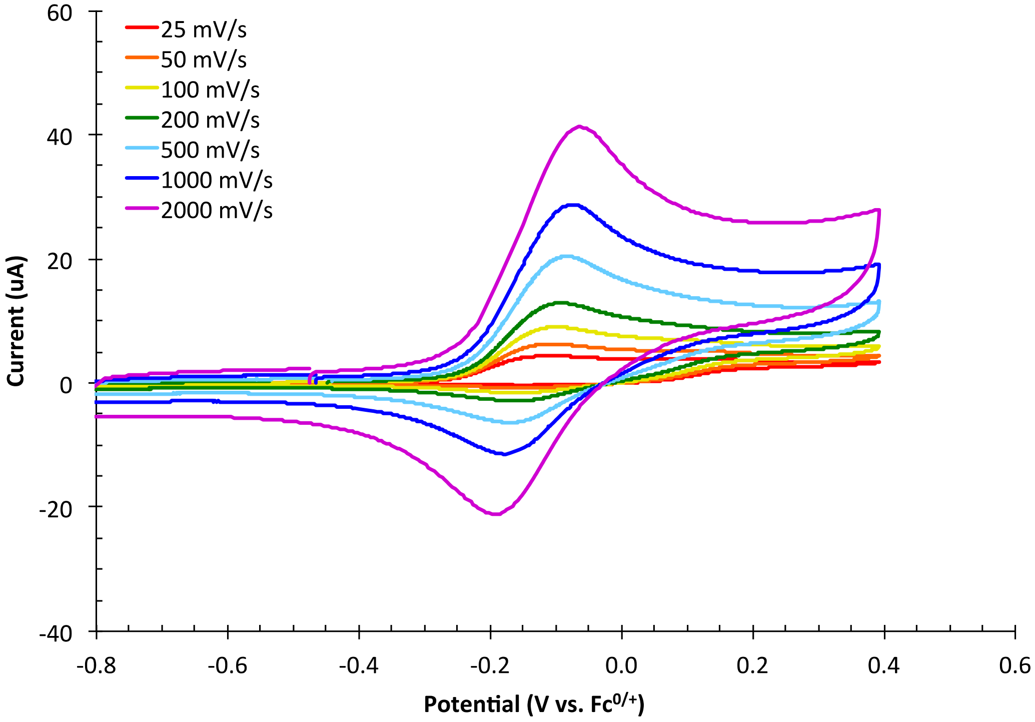

Figure S20. Cyclic voltammograms of 1a (1.0 mM) in the presence of Et-P_2 (1.2 mM) in 0.1 M Bu_4NClO_4 in DMSO at various scan rates. Anodic sweep.

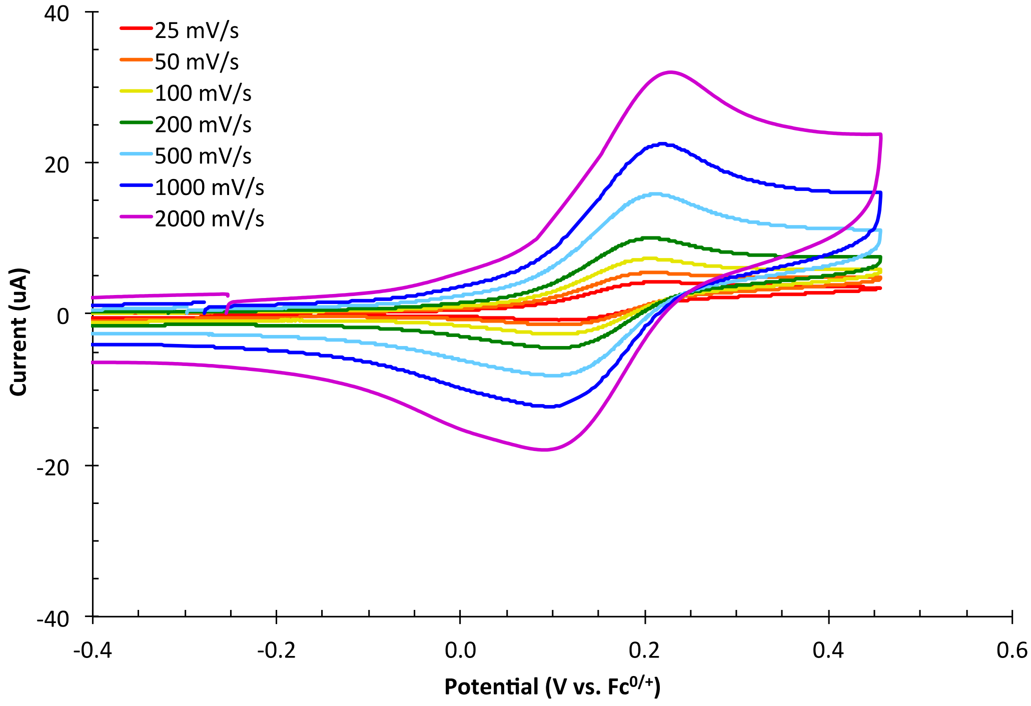

Figure S21. Cyclic voltammograms of **1c** (1.0 mM) in 0.1 M Bu_4NClO_4 in DMSO at various scan rates. Anodic sweep.

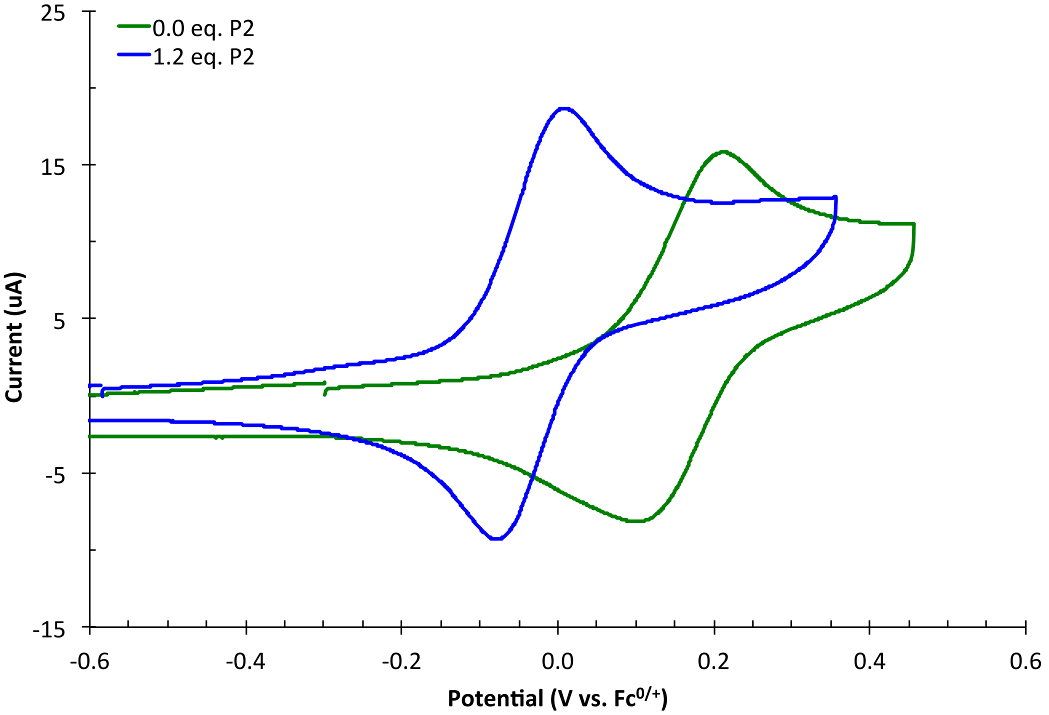

Figure S22. Cyclic voltammograms of 1c (1.0 mM) in the absence (green trace) and presence of Et-P_2 (1.2 mM, blue trace) in 0.1 M Bu_4NClO_4 in DMSO. Scan rate 500 mV/s.

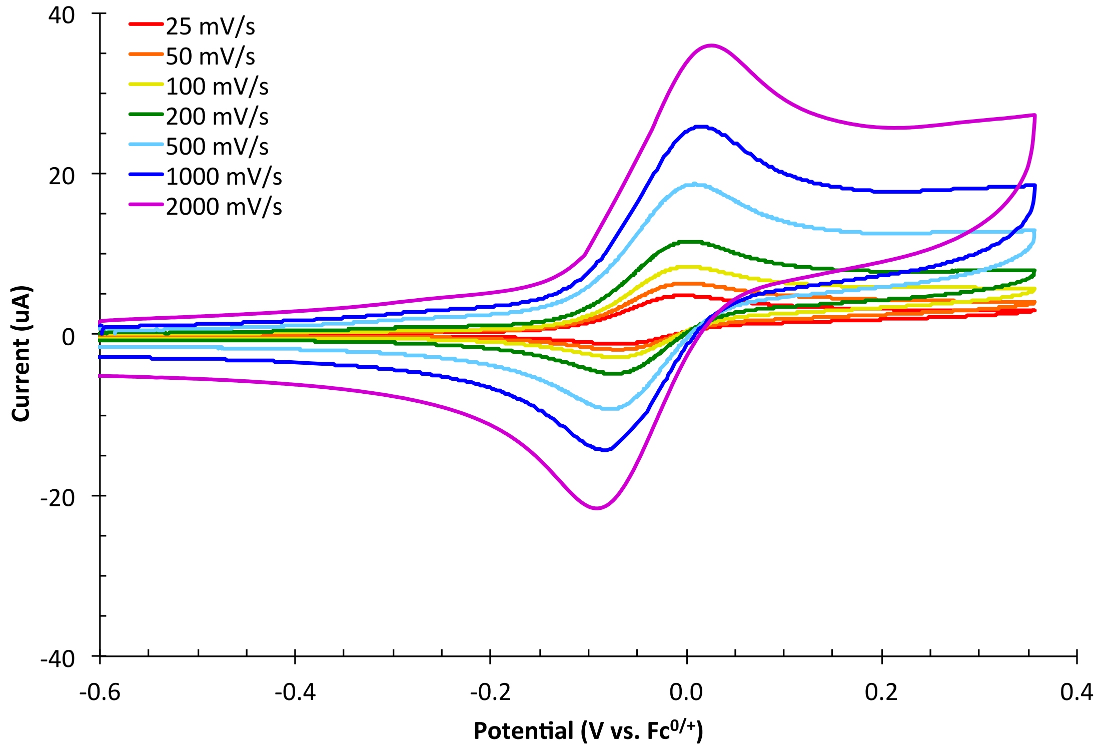

Figure S23. Cyclic voltammograms of **1c** (1.0 mM) in the presence of Et-P_2 (1.2 mM) in 0.1 M Bu_4NClO_4 in DMSO at various scan rates. Anodic sweep.

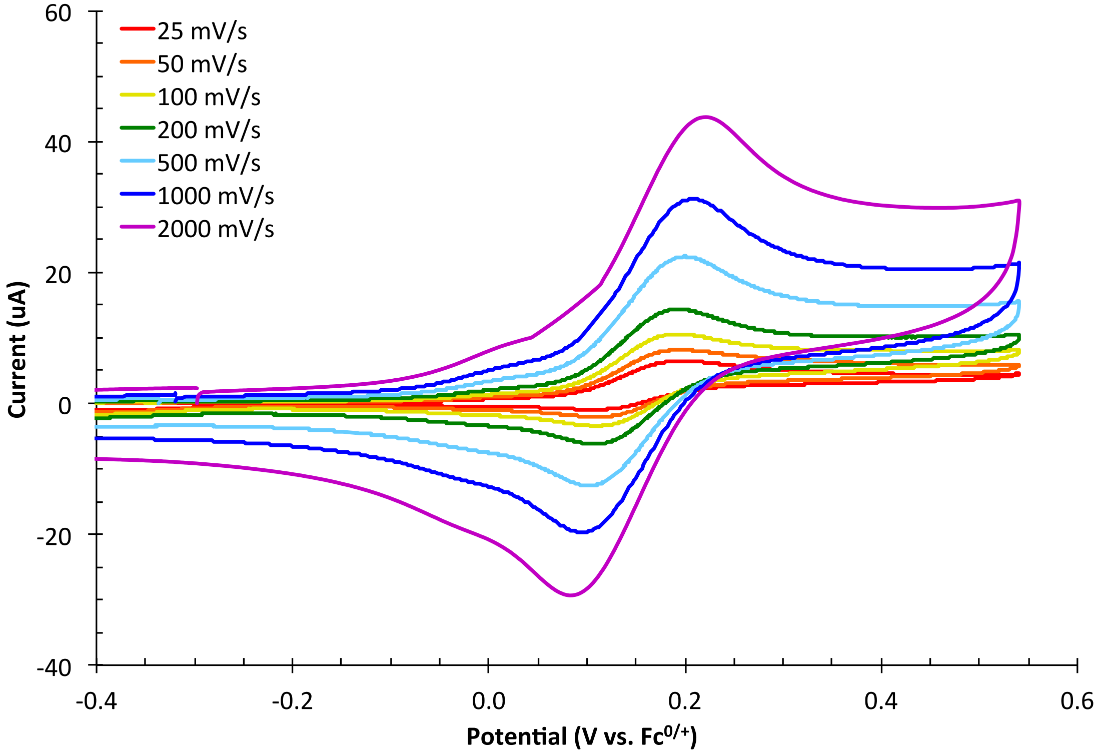

Figure S24. Cyclic voltammograms of 1b (1.0 mM) in 0.1 M Bu_4NClO_4 in DMSO at various scan rates. Anodic sweep.

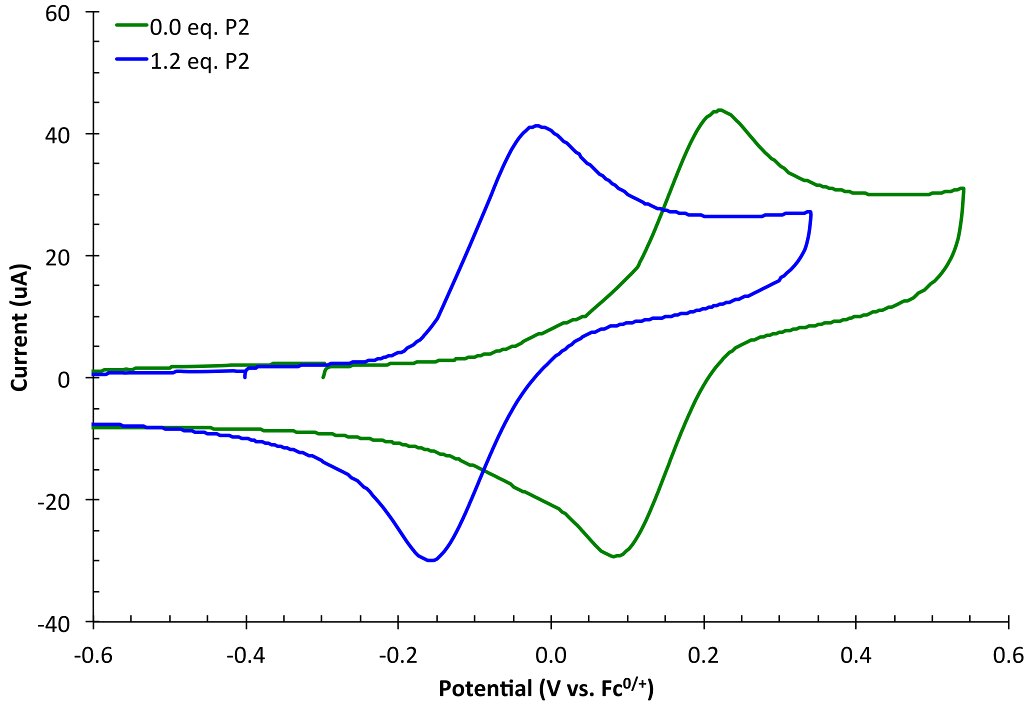

Figure S25. Cyclic voltammograms of **1b** (1.0 mM) in the absence (green trace) and presence of Et-P_2 (1.2 mM, blue trace) in 0.1 M Bu_4NClO_4 in DMSO. Scan rate 2000 mV/s.

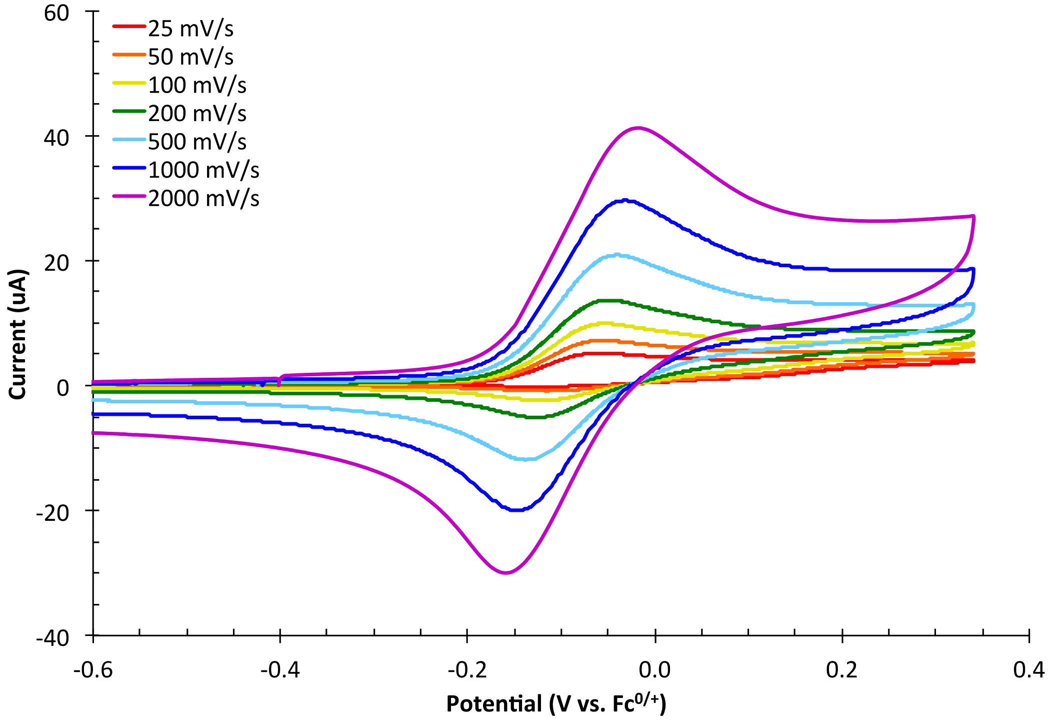

Figure S26. Cyclic voltammograms of **1b** (1.0 mM) in the presence of Et-P_2 (1.2 mM) in 0.1 M Bu_4NClO_4 in DMSO at various scan rates. Anodic sweep.

Determination of pK_a Values

Square Scheme Analysis to Determine ΔpK$_a$

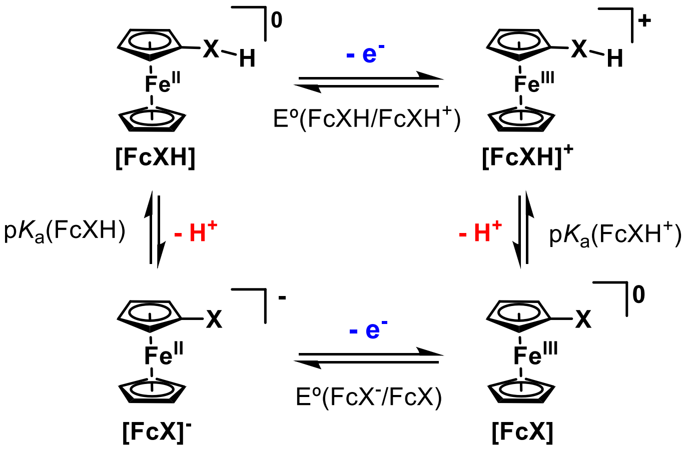

Figure S27. Square scheme for FcXH species.

$$\Delta G_{PT}(FcXH/FcX^{-}) + \Delta G_{ET}(FcX^{-}/FcX) = \Delta G_{ET}(FcXH/FcXH^{+}) + \Delta G_{PT}(FcXH^{+}/FcX) \quad (1)$$

$$\Delta G_{PT} = -RT\ln(K_{eq}) = 2.303RT \times pK_a \quad (2)$$

$$\Delta G_{ET} = -nFE^{o} = 23.06\,E^{o} \quad (3)$$

$$2.303\mathrm{RTp}K_a(FcXH) + 23.06E^{o}(FcX^{-}/FcX) = 23.06E^{o}(FcXH/FcXH^{+}) + 2.303\mathrm{RTp}K_a(FcXH^{+}) \quad (4)$$

$$2.303\mathrm{RT} \times [\mathrm{p}K_a(FcXH) - \mathrm{p}K_a(FcXH^{+})] = 23.06 \times [E^{o}(FcXH/FcXH^{+}) - E^{o}(FcX^{-}/FcX)] \quad (5)$$

$$2.303\mathrm{RT} \times \Delta\mathrm{p}K_a = 23.06 \times \Delta E^{o} \quad (6)$$

$$\Delta\mathrm{p}K_a = \frac{23.06}{2.303RT} \times \Delta E^{o} \quad (7)$$

Table S7. Redox potentials of FcXH species **1a**, **1b**, **1c**, and **2a**.

	$E_{1/2}(FcXH/FcXH^+)$[a] V vs. $Fc^{0/+}$	$E_{1/2}(FcX^- /FcX)$[a] V vs. $Fc^{0/+}$	$\Delta E_{1/2}$[d]	ΔpK_a[e]
$Fc(CO_2H)$[b] (**2a**)	0.208	–0.095	0.303	5.1
$FcP(O)(OH)_2$[b] (**1a**)	0.114	–0.126	0.240	4.0
FcP(O)(OPh)(OH)[c] (**1c**)	0.158	–0.032	0.190	3.2
FcP(O)(Ph)(OH)[b] (**1b**)	0.153	–0.090	0.243	4.1

[a] $E_{1/2} = 0.5(E_{pa} + E_{pc})$, where E_{pa} and E_{pc} are anodic and cathodic peak potentials, respectively. [b] E_{pa} and E_{pc} taken at 2000 mv/s. [c] E_{pa} and E_{pc} taken at 500 mv/s. [d] $\Delta E_{1/2} = E_{1/2}(FcXH/FcXH^+) - E_{1/2}(FcX^-/FcX)$. [e] Determined via Equation 7.

General Procedure for pK_a Titrations

In an inert atmosphere glovebox, a 15 mM solution of XH in 0.6 mL DMSO-d_6 was prepared in a septum-capped NMR tube with dichloromethane (0.05 mL of a 0.15 M stock solution in DMSO-d_6) added as an internal standard. A stock solution of base (0.20 M in DMSO-d_6) was prepared and aliquots were added to the NMR tube. The NMR solution was allowed to equilibrate for three min before a ^{1}H NMR spectrum was acquired. All ^{1}H NMR spectra were recorded on a 600 MHz spectrometer with a relaxation delay (d_1) of 20 s.

Upon addition of base, an equilibrium mixture of the protonated (XH, *e.g.* FcP(O)$(OH)_2$) and deprotonated ($X^–$, *e.g.* FcP(O)(OH)($O^–$) is rapidly established. As a result, only one set of ^{1}H NMR resonances is observed, and it corresponds to the weighted average of spectrum of XH and $X^–$. Similarly, a weighted average spectrum of the base (B, *e.g.* triethylamine) and its conjugate acid (BH^+, *e.g.* triethylammonium) is observed. The mol fraction (χ) of each species can be determined by the chemical shift of the resonances attributed to XH/$X^–$ and the chemical shift of the resonances attributed to B/BH^+. From this data, the equilibrium constant K_{eq} of the reaction can be calculated and the pK_a of XH can be determined provided that the pK_a of BH^+ is known. All ^{1}H NMR titrations were performed in duplicate. Because the duplicates were within error of each other, the reported pK_a value is the average value obtained from both titrations. Additional information detailing the theory and procedure for pK_a determination by NMR titration can be found in references 5-9.

$$\chi_{FcX^-} = \frac{chemical\ shift_{FcXH} - chemical\ shift_{observed}}{chemical\ shift_{FcXH} - chemical\ shift_{FcX^-}} \quad (8)$$

$$\chi_{FcXH} = 1 - x_{FcX^-} \quad (9)$$

$$\chi_{BH^+} = \frac{chemical\ shift_{B} - chemical\ shift_{observed}}{chemical\ shift_{B} - chemical\ shift_{BH^+}} \quad (10)$$

$$\chi_{B} = 1 - x_{BH^+} \quad (11)$$

$$K_{eq} = \frac{\chi_{FcX^-}\chi_{BH^+}}{\chi_{FcXH}\chi_{B}} \quad (12)$$

$$K_{eq} = \frac{K_a(FcXH)}{K_a(BH^+)} \quad (13)$$

$$K_a(FcXH) = K_{eq}K_a(BH^+) \quad (14)$$

$$pK_a(FcXH) = -log\big(K_a(FcXH)\big) \quad (15)$$

Table S8. pK_a values of relevant acids in DMSO.

	pK_a (XH)[a]	ΔpK_a[b]	pK_a (XH^+)[c]
Fc(CO_2H)[d] (**2a**)	10.8 ± 0.2	5.1	5.7 ± 0.2
FcP(O)$(OH)_2$[e] (**1a**)	8.6 ± 0.2	4.0	4.6 ± 0.2
FcP(O)(OPh)(OH)[e] (**1c**)	7.2 ± 0.3	3.2	4.0 ± 0.3
FcP(O)(Ph)(OH)[e] (**1b**)	8.5 ± 0.4	4.1	4.4 ± 0.4
P(O)$(OPh)_2$(OH)[f]	3.7 ± 0.2	--	--
P(O)(Ph)$(OH)_2$[e]	8.0 ± 0.2	--	--

[a] Determined through ^{1}H NMR titration. [b] Determined through square-scheme analysis using electrochemical data in Table S and Equation 7. [c] pK_a (XH^+) = pK_a (XH) - ΔpK_a. [d] Titration performed with pyrrolidine. [e] Titration performed with triethylamine. [f] Titration performed with pyridine.

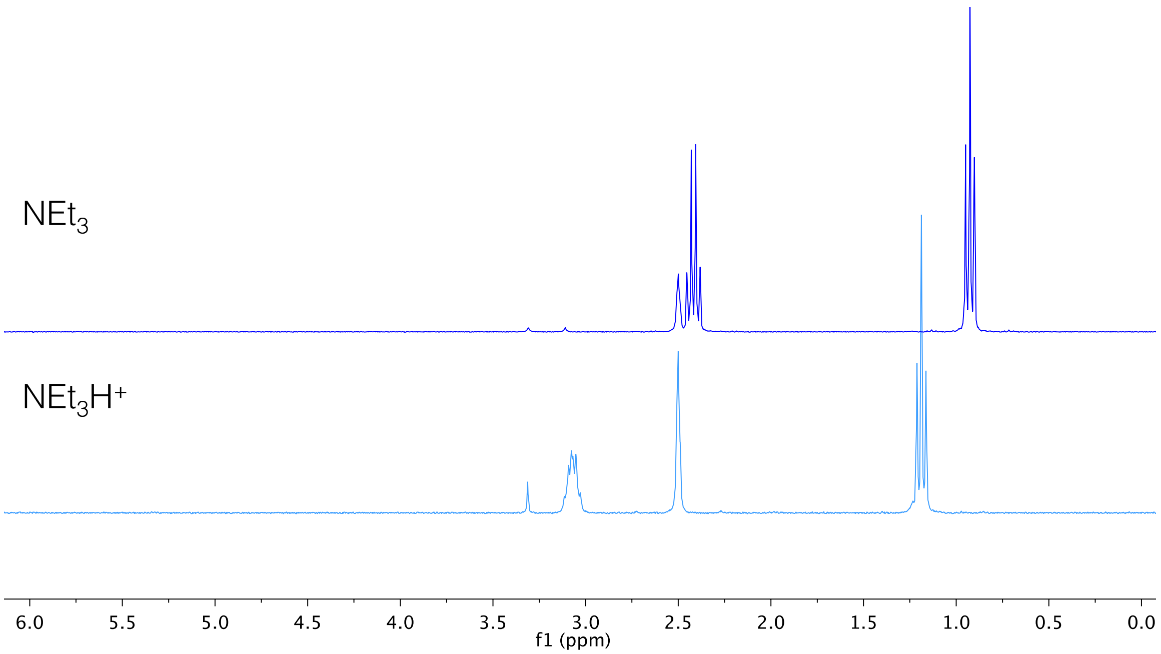

Figure S28. ^{1}H NMR spectra of triethylamine (NEt_3, top spectrum, dark blue) and triethylamine hydrochloride (NEt_3HCl, bottom spectrum, light blue) in DMSO-d_6.

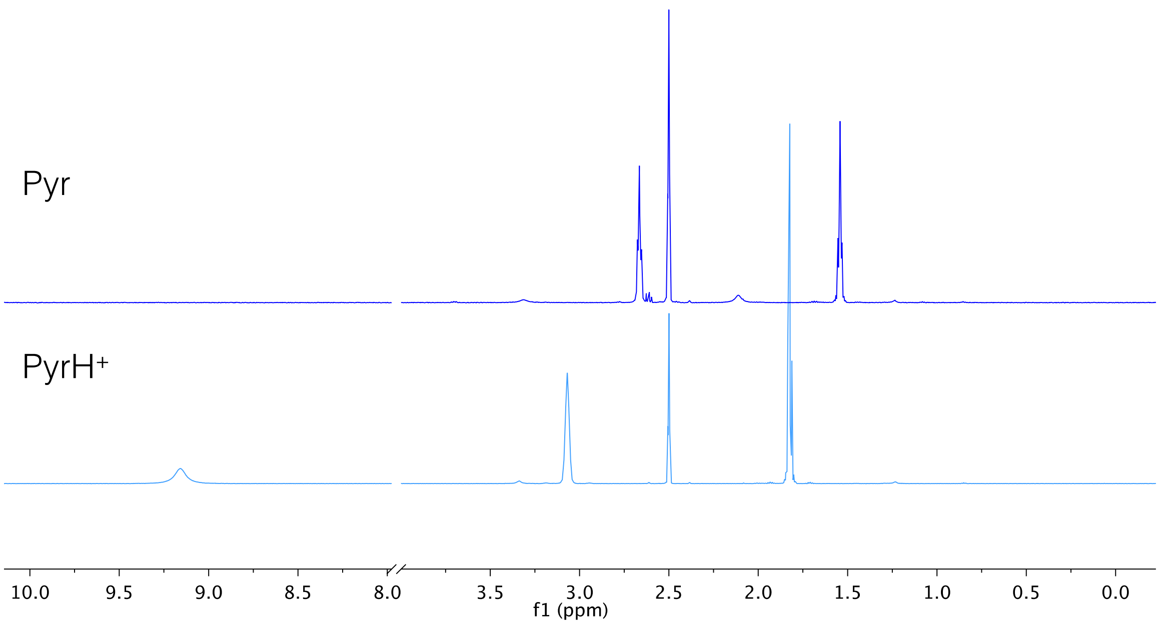

Figure S29. ^{1}H NMR spectra of pyrrolidine (Pyr, top spectrum, dark blue) and pyrrolidine hydrochloride ($PyrH^+$, bottom spectrum, light blue) in DMSO-d_6.

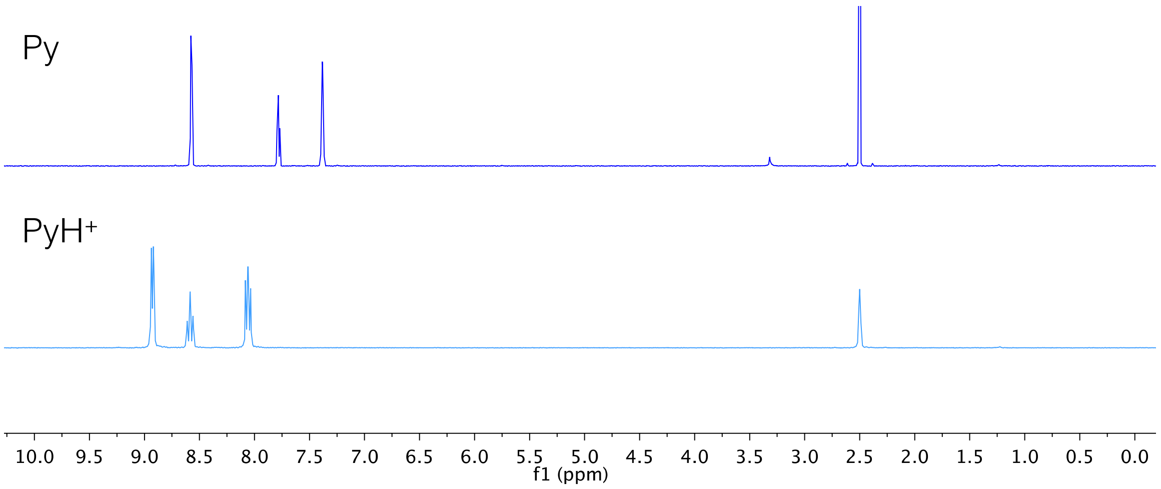

Figure S30. ^{1}H NMR spectra of pyridine (Py, top spectrum, dark blue) and pyridine hydrochloride (PyH^+, bottom spectrum, light blue) in DMSO-d_6.

pK_a Titration of Fc(CO_2H) ***2a*** *with pyrrolidine (Pyr)*

Chemical shift analysis of the resonances at 4.46-4.76 ppm, 4.13-4.50 ppm, and 4.07-4.26 ppm yielded an average value for the mol fractions of Fc(CO_2H) **2a** (χFc(CO_2H)) and its conjugate base Fc(CO_2^-) (χFc(CO_2^-)).The mol fractions of pyrrolidine and its conjugate acid pyrrolidinium (χPyr and χPyrH$^+$, respectively) were determined by chemical analysis of the resonance at 1.54-1.82 ppm.

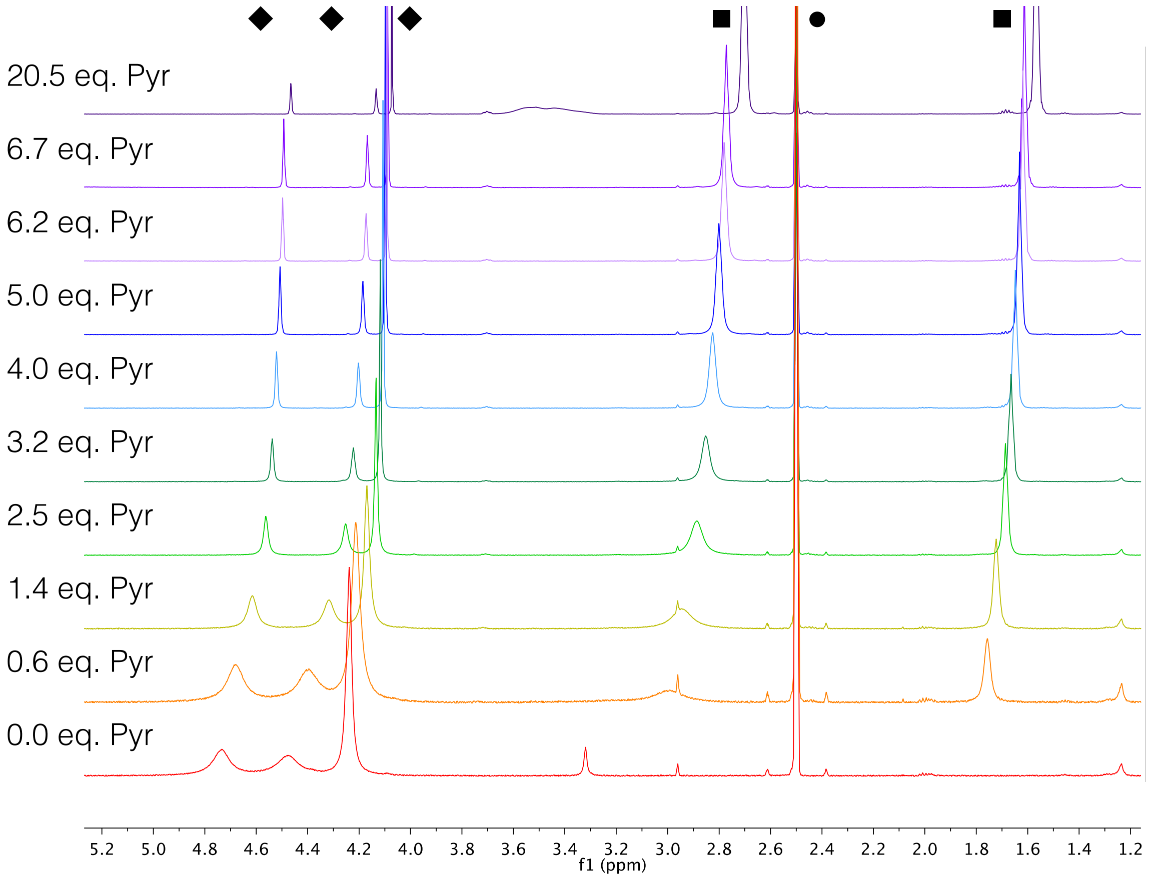

Figure S31. ^{1}H NMR titration of Fc(CO_2H) **2a** in DMSO-d_6 with pyrrolidine (Pyr, 0.20 M stock solution in DMSO-d_6). DMSO is noted with a circle (●), resonances attributed to Fc(CO_2H)/Fc(CO_2)$^-$ are noted with diamonds (◆), and resonances attributed to pyrrolidine/pyrrolidinium (PyrH$^+$) are noted with squares (■).

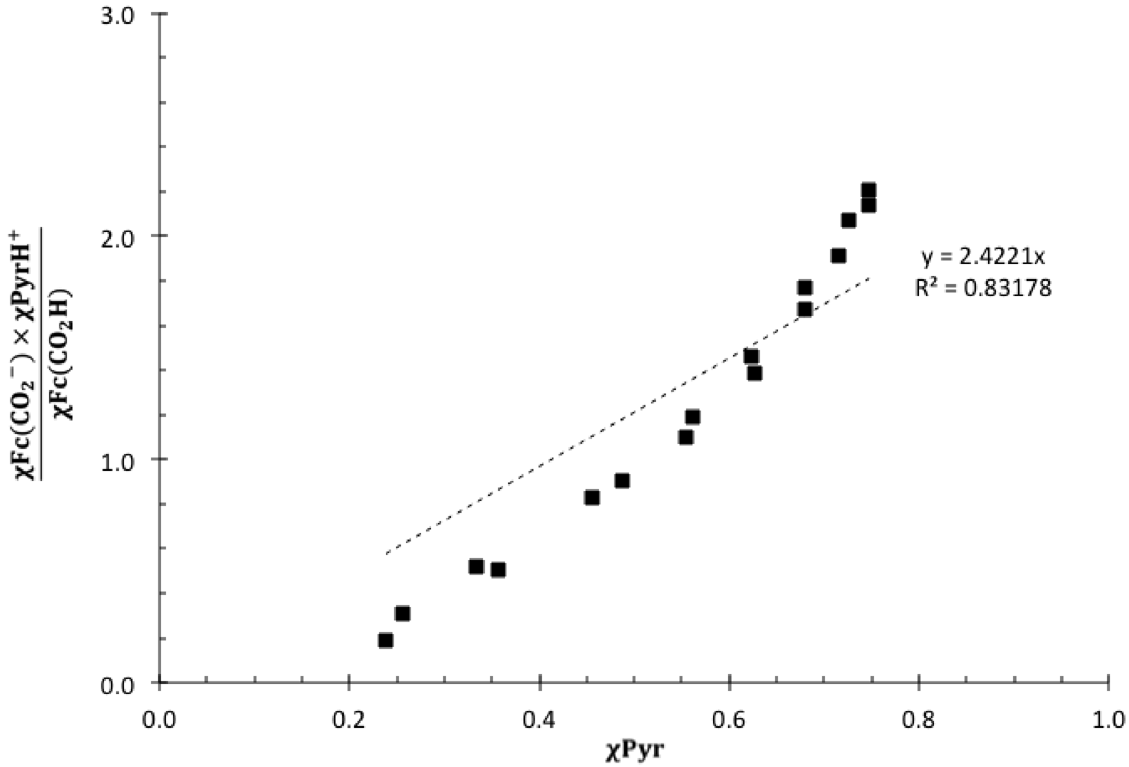

Figure S32. Plot of [χFc(CO_2)$^-$ × χ($PyrH^+$)]/[χFc(CO_2H)] versus [χPyr] where χ is the mole fraction as determined by chemical shift. When the y-intercept is set to 0, the slope of the linear regression yields K_{eq}.

*pK_a Titration of FcP(O)(OH)$_2$ **1a** with triethylamine (NEt$_3$)*

Chemical shift analysis of the resonances at 4.29-4.35 ppm and 4.25-4.21 ppm yielded an average value for the mol fractions of FcP(O)(OH)$_2$ **1a** (χ FcP(O)(OH)$_2$) and its conjugate base FcP(O)(OH)(O$^-$) (χFcP(O)(OH)(O$^-$)). The mol fractions of triethylamine and its conjugate acid triethylammonium (χNEt$_3$ and χNEt$_3$H$^+$, respectively) were determined by chemical analysis of the resonance at 0.93-1.19 ppm.

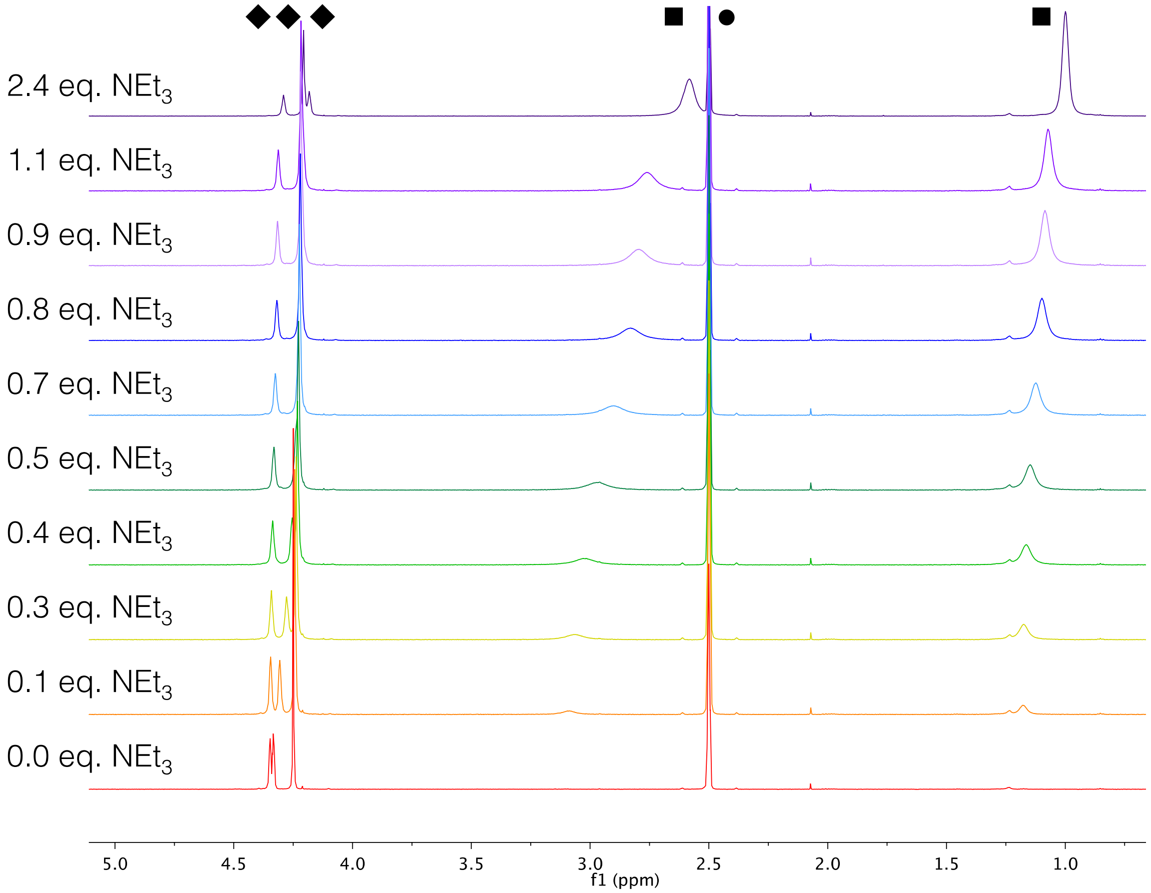

Figure S33. ^{1}H NMR titration of FcP(O)(OH)$_2$ **1a** in DMSO-d$_6$ with triethylamine (NEt$_3$) (0.20 M stock solution in DMSO-d$_6$). DMSO is noted with a circle (●), resonances attributed to FcP(O)(OH)$_2$/FcP(O)(OH)(O$^-$) are noted with diamonds (◆), and resonances attributed to NEt$_3$/NEt$_3$H$^+$ are noted with squares (■).

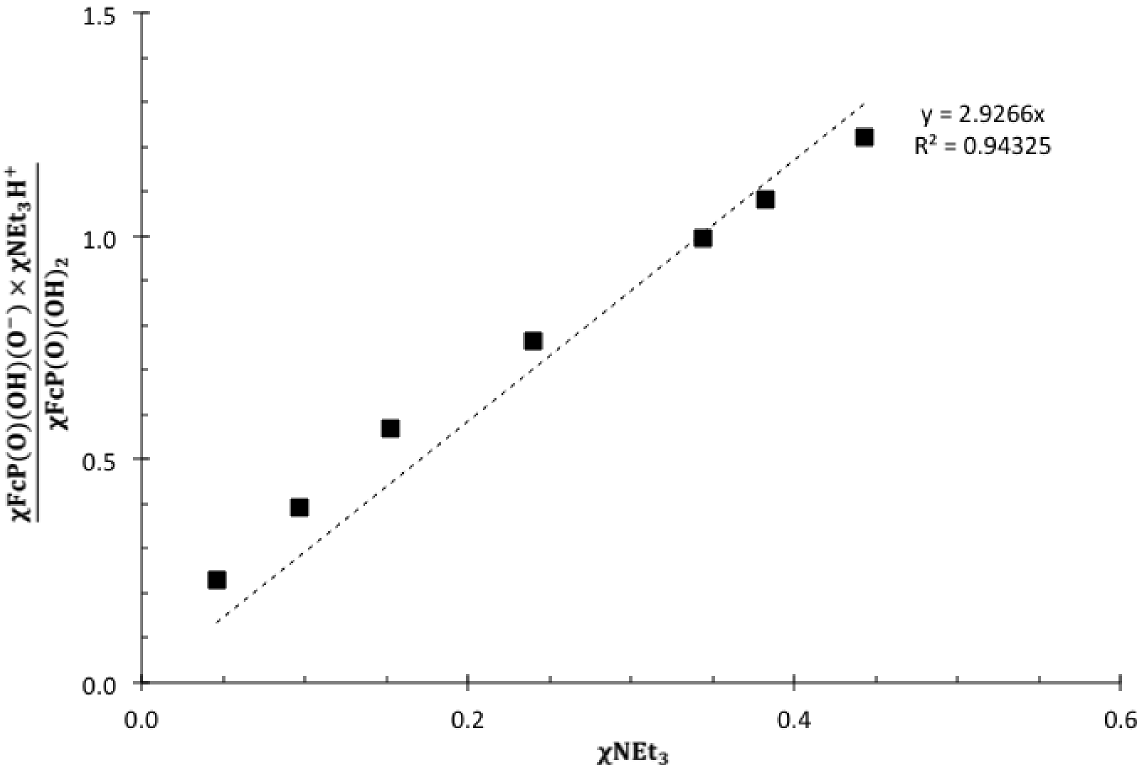

Figure S34. Plot of [χFcP(O)(OH)(O$^-$) × χ(NEt_3H^+)]/[χFcP(O)$(OH)_2$] versus [χNEt_3] where χ is the mole fraction as determined by chemical shift analysis. When the y-intercept is set to 0, the slope of the linear regression yields K_{eq}.

pK_a Titration of FcP(O)(OPh)(OH) ***1c*** *with triethylamine (NEt_3)*

Chemical shift analysis of the resonances at 7.15-7.32 ppm, 6.88-7.10 ppm, and 4.15-4.26 ppm yielded an average value for the mol fractions of FcP(O)(OPh)(OH) **1c** (χFcP(O)(OPh)(OH)) and its conjugate base FcP(O)(OPh)(O^-) (χFcP(O)(OPh)(O^-)). The mol fractions of triethylamine and its conjugate acid triethylammonium (χNEt_3 and χNEt_3H^+, respectively) were determined by chemical analysis of the resonance at 2.42-3.07 ppm.

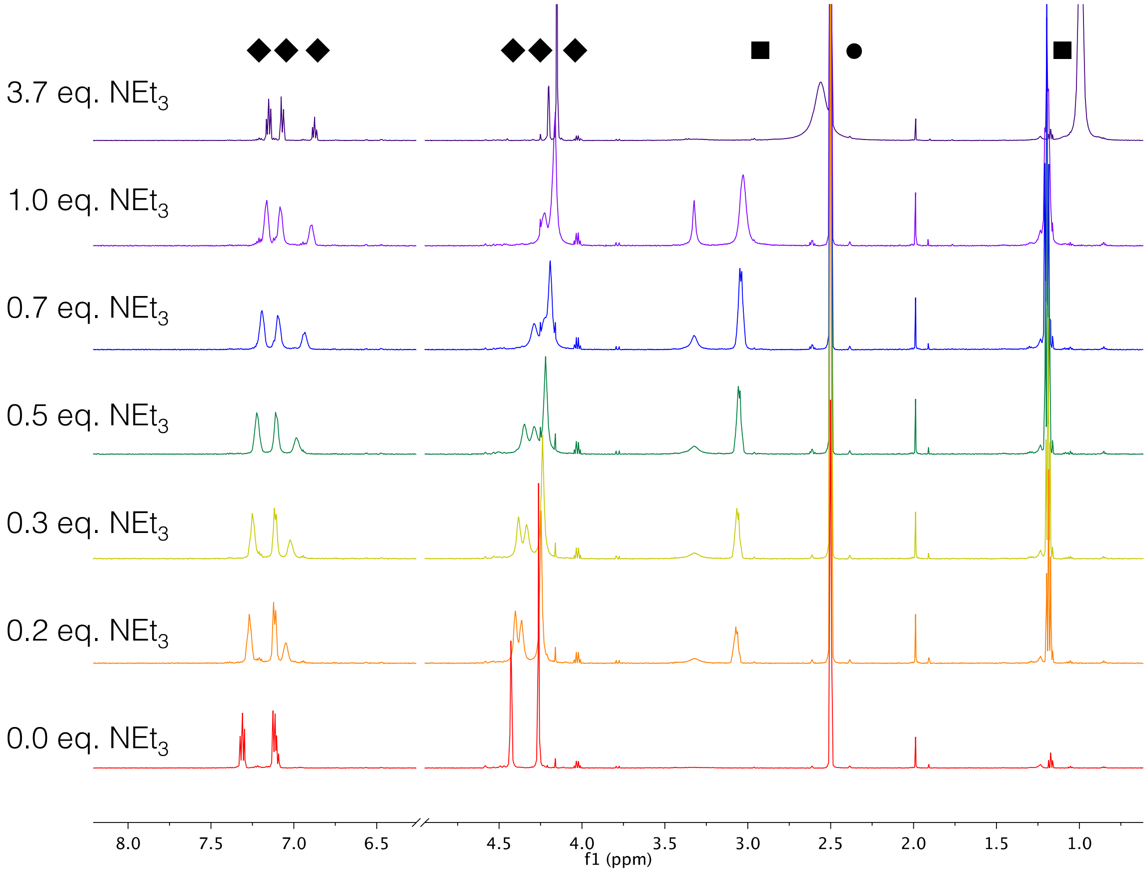

Figure S35. ^1H NMR titration of FcP(O)(OPh)(OH) **1c** in DMSO-d_6 with triethylamine (NEt_3) (0.20 M stock solution in DMSO-d_6). DMSO is noted with a circle (●), resonances attributed to FcP(O)(OPh)(OH)/FcP(O)(OPh)(O^-) are noted with diamonds (◆), and resonances attributed to NEt_3/NEt_3H^+ are noted with squares (■).

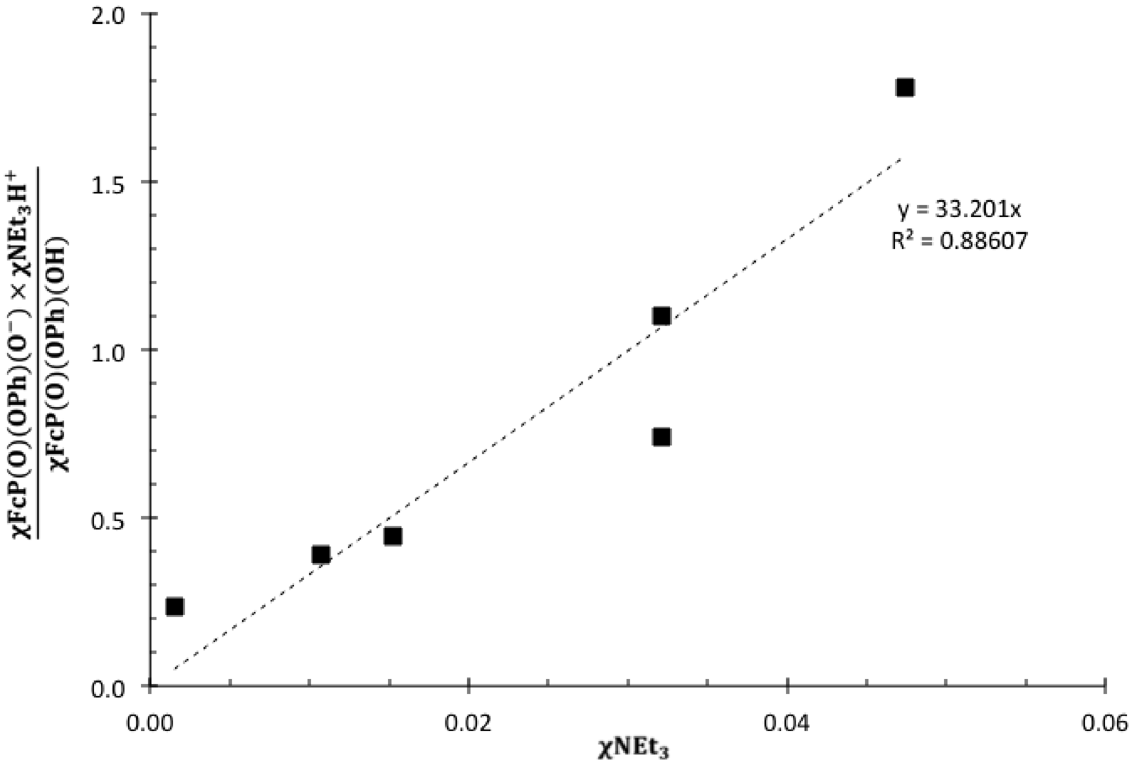

Figure S36. Plot of [χFcP(O)(OPh)(O^{-}) × χ(NEt_3H^+)]/[χFcP(O)(OPh)(OH)] versus [χNEt_3] where χ is the mole fraction as determined by chemical shift analysis. When the y-intercept is set to 0, the slope of the linear regression yields K_{eq}.

pK_a Titration of FcP(O)(Ph)(OH) ***1b*** *with triethylamine (NEt_3)*

Chemical shift analysis of the resonances at 7.67-7.72 ppm, 7.28-7.46 ppm, and 4.09-4.20 ppm yielded an average value for the mol fractions of FcP(O)(Ph)(OH) (χFcP(O)(Ph)(OH)) and its conjugate base FcP(O)(Ph)(O^-) (χFcP(O)(Ph)(O^-)). The mol fractions of triethylamine and its conjugate acid triethylammonium (χNEt_3 and χNEt_3H^+, respectively) were determined by chemical analysis of the resonance at 0.93-1.19 ppm.

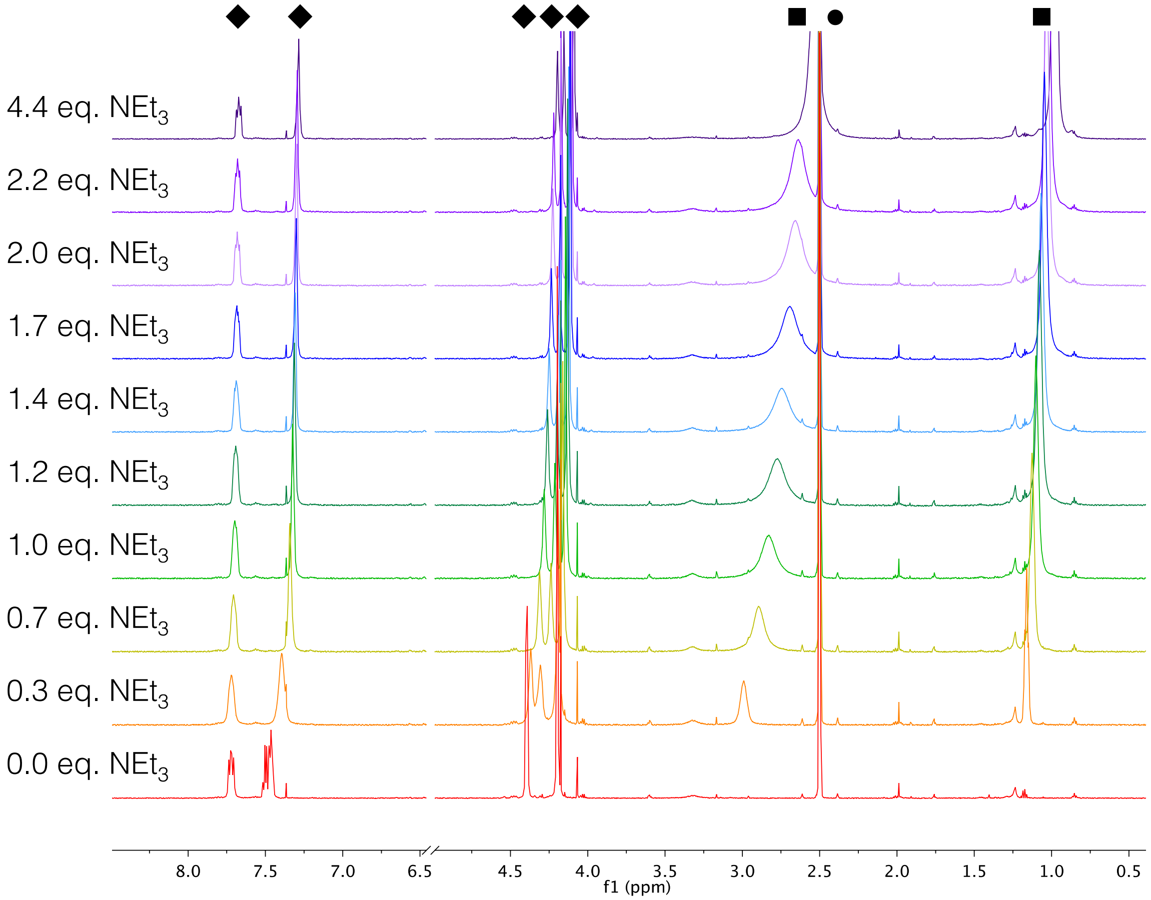

Figure S37. ^{1}H NMR titration of FcP(O)(Ph)(OH) 1b in DMSO-d_6 with triethylamine (NEt_3) (0.20 M stock solution in DMSO-d_6). DMSO is noted with a circle (●), resonances attributed to FcP(O)(Ph)(OH)/FcP(O)(Ph)(O^-) are noted with diamonds (◆), and resonances attributed to NEt_3/NEt_3H^+ are noted with squares (■).

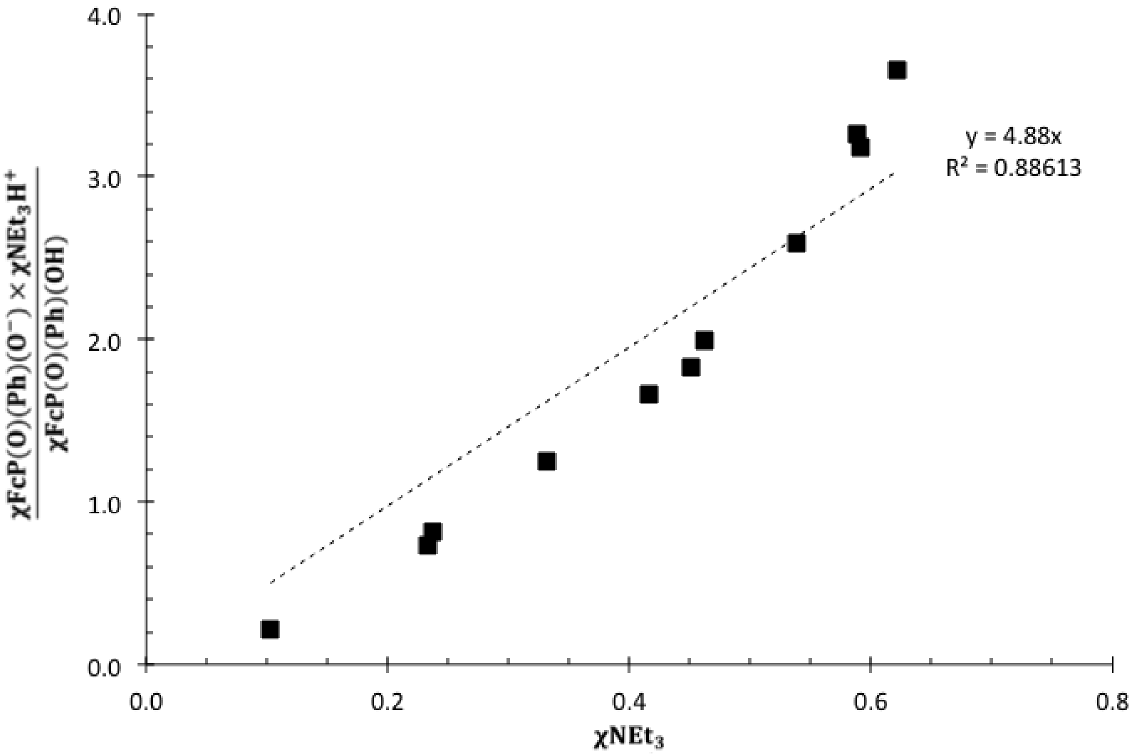

Figure S38. Plot of [χFcP(O)(Ph)(O^{-}) × χ(NEt_3H^+)]/[χFcP(O)(Ph)(OH)] versus [χNEt_3] where χ is the mole fraction as determined by chemical shift analysis. When the y-intercept is set to 0, the slope of the linear regression yields K_{eq}.

pK_a Titration of $P(O)(OPh)_2(OH)$ with pyridine (Py)

Chemical shift analysis of the resonances at 7.26-7.37 ppm and 7.01-7.17 ppm yielded an average value for the mol fractions of $P(O)(OPh)_2(OH)$ ($\chi P(O)(OPh)_2(OH)$) and its conjugate base $P(O)(OPh)_2(O)^-$ ($\chi P(O)(OPh)_2(O)^-$). The average values for the mol fractions of pyridine and its conjugate acid pyridinium (χPy and χPyH^+, respectively) were determined by chemical analysis of the resonances at 8.57-8.93 ppm and 7.78-8.59 ppm.

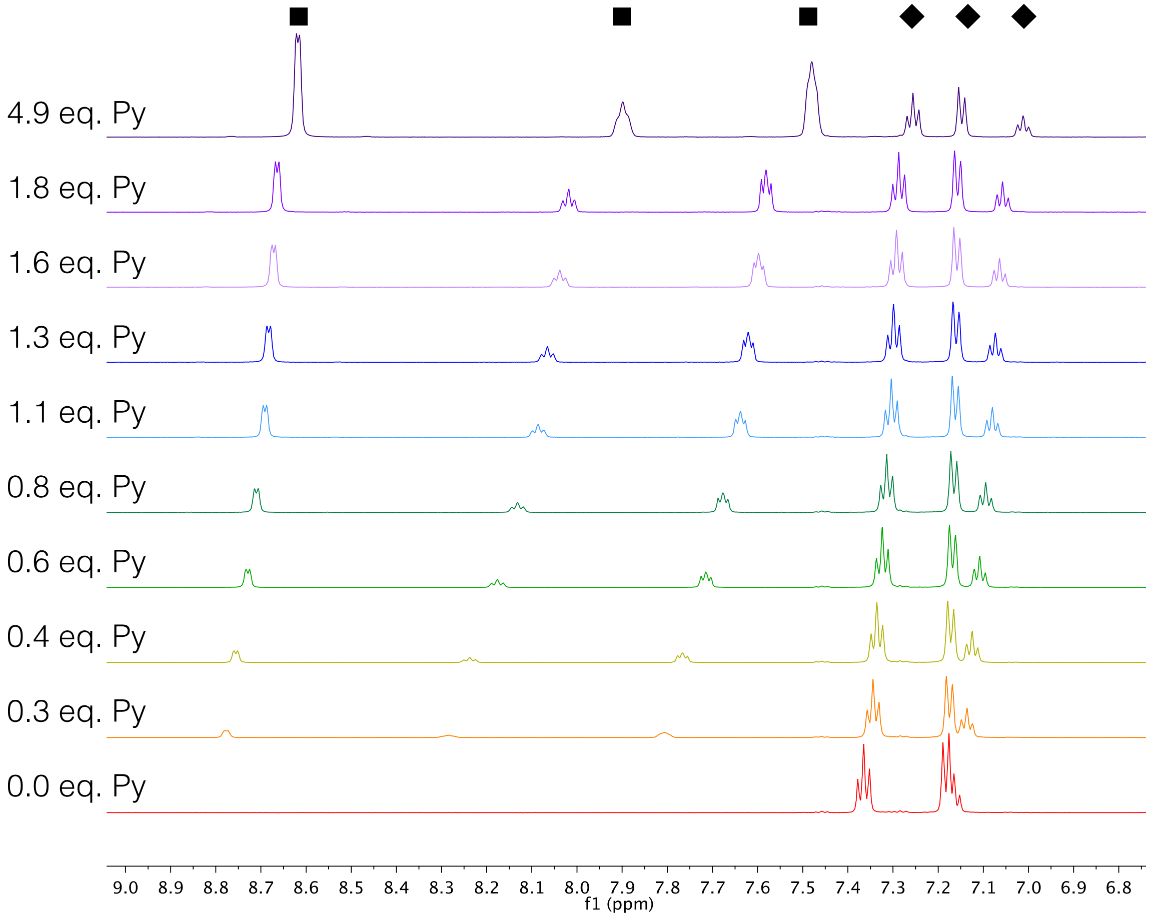

Figure S39. ^{1}H NMR titration of $P(O)(OPh)_2(OH)$ in DMSO-d_6 with pyridine (Py) (0.20 M stock solution in DMSO-d_6). Resonances attributed to $P(O)(OPh)_2(OH)$/ $P(O)(OPh)_2(O)^-$ are noted with diamonds (◆), and resonances attributed to pyridine/pyridinium (PyH^+) are noted with squares (■).

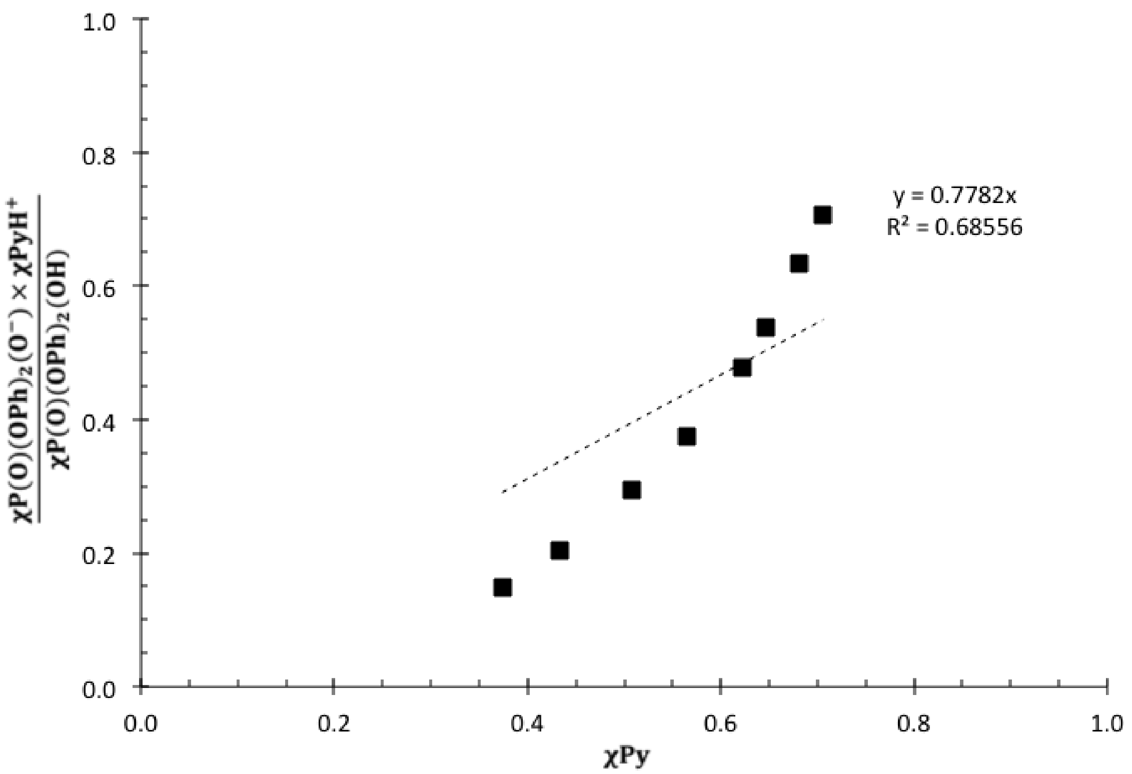

Figure S40. Plot of [χP(O)(OPh)$_2$(O)$^-$ × χ(PyH$^+$)]/[χP(O)(OPh)$_2$(OH)] versus [χPy] where χ is the mole fraction as determined by chemical shift analysis. When the y-intercept is set to 0, the slope of the linear regression yields K_{eq}.

pK_a Titration of $P(O)(Ph)(OH)_2$ with triethylamine (NEt_3)

Chemical shift analysis of the resonances at 7.68-7.62 ppm and 7.29-7.45 ppm yielded an average value for the mol fractions of $P(O)(Ph)(OH)_2$ ($\chi P(O)(Ph)(OH)_2$) and its conjugate base $P(O)(Ph)(OH)(O^-)$ ($\chi P(O)(Ph)(OH)(O^-)$). The mol fractions of triethylamine and its conjugate acid triethylammonium (χNEt_3 and χNEt_3H^+, respectively) were determined by chemical analysis of the resonance at 0.93-1.19 ppm.

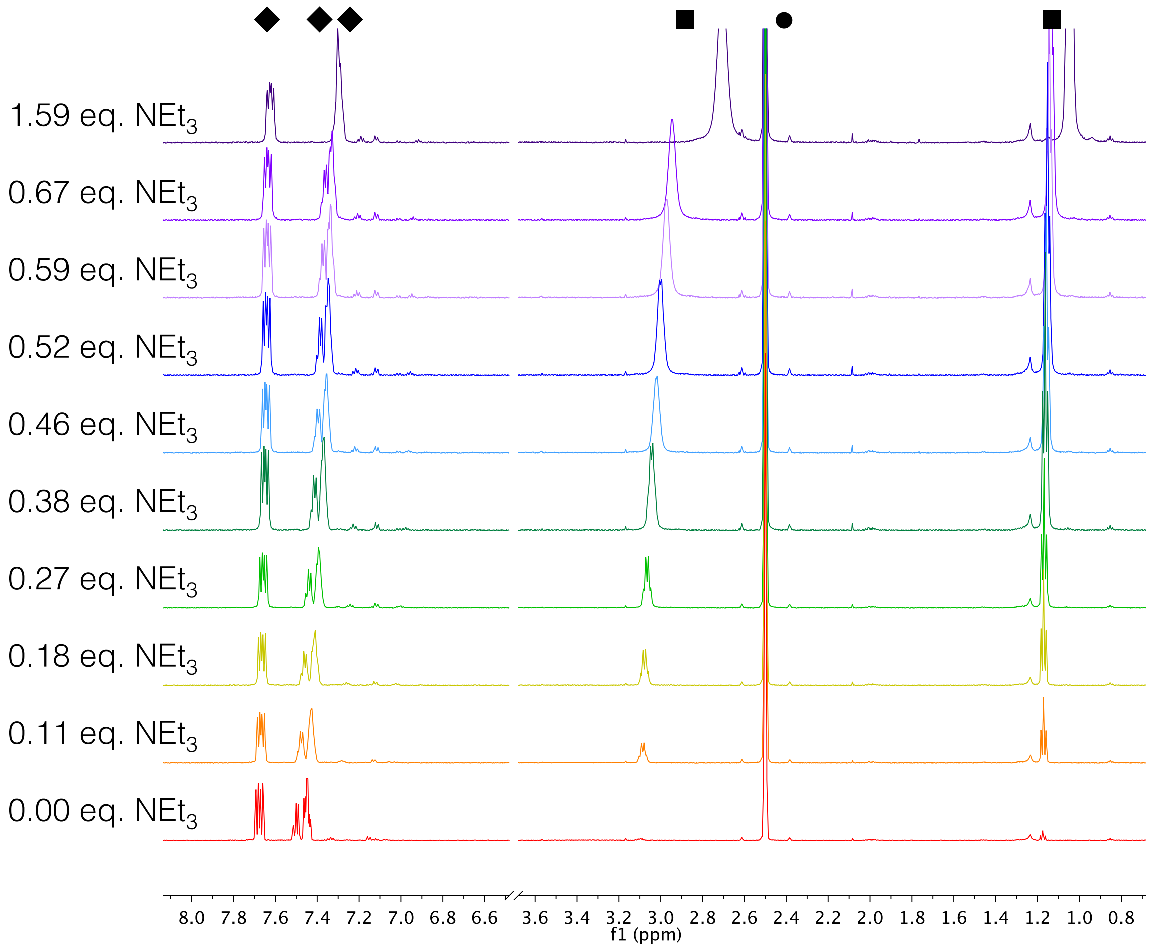

Figure S41. ^1H NMR titration of $P(O)(Ph)(OH)_2$ in DMSO-d_6 with triethylamine (NEt_3) (0.20 M stock solution in DMSO-d_6). DMSO is noted with a circle (●), resonances attributed to $P(O)(Ph)(OH)_2$/$P(O)(Ph)(OH)(O^-)$ are noted with diamonds (◆), and resonances attributed to NEt_3/NEt_3H^+ are noted with squares (■).

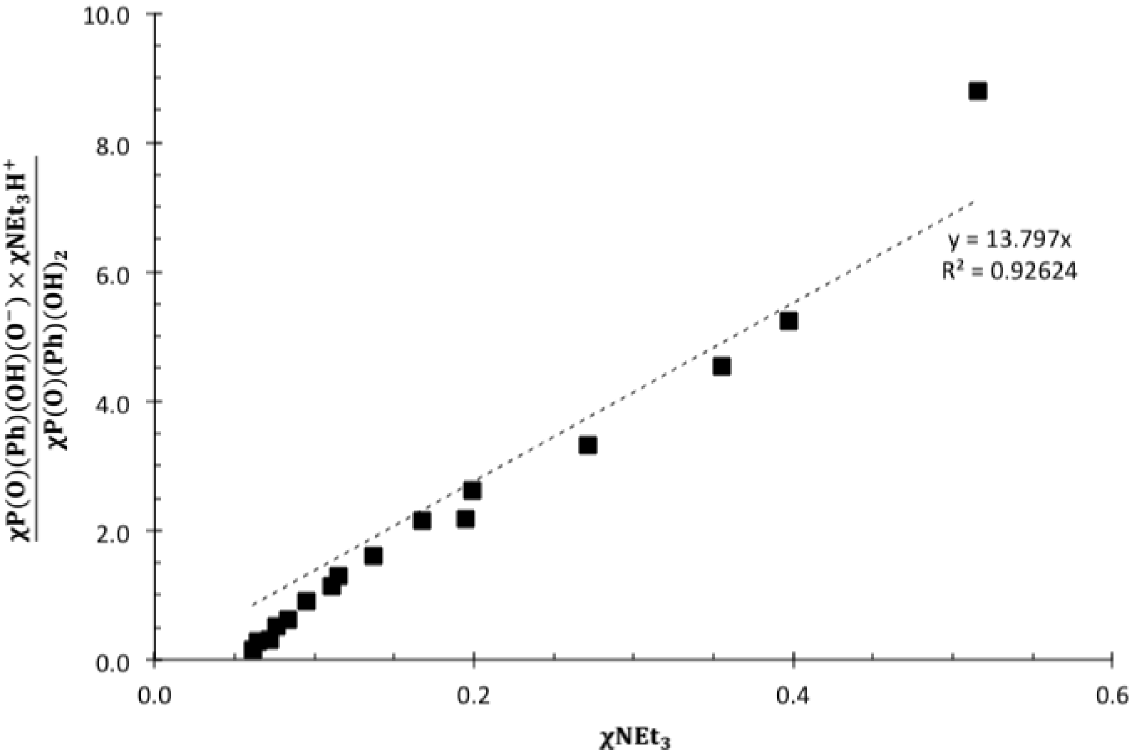

Figure S42. Plot of [χP(O)(Ph)(OH)(O)$^-$ × χNEt_3H^+]/[χP(O)(Ph)$(OH)_2$] versus [χNEt_3] where χ is the mole fraction as determined by chemical shift analysis. When the y-intercept is set to 0, the slope of the linear regression yields K_{eq}.

NMR spectra

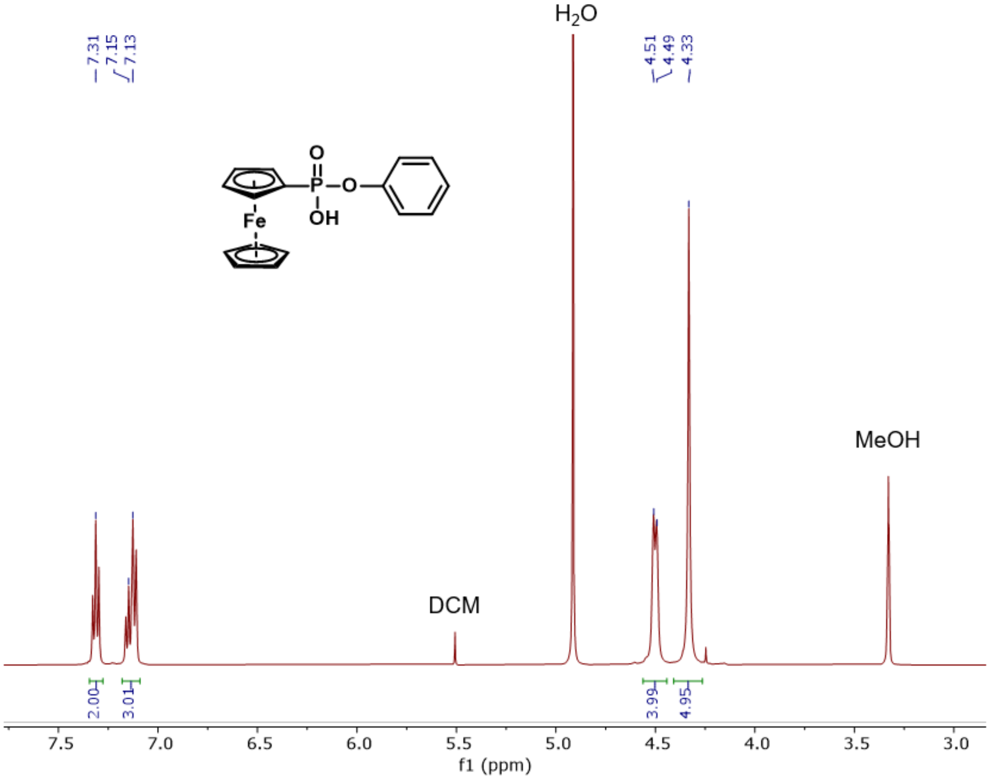

Figure S43. ^{1}H NMR spectrum acquired in CD_3OD of ferrocenyl (phenyl)phosphonic acid (**1c**).

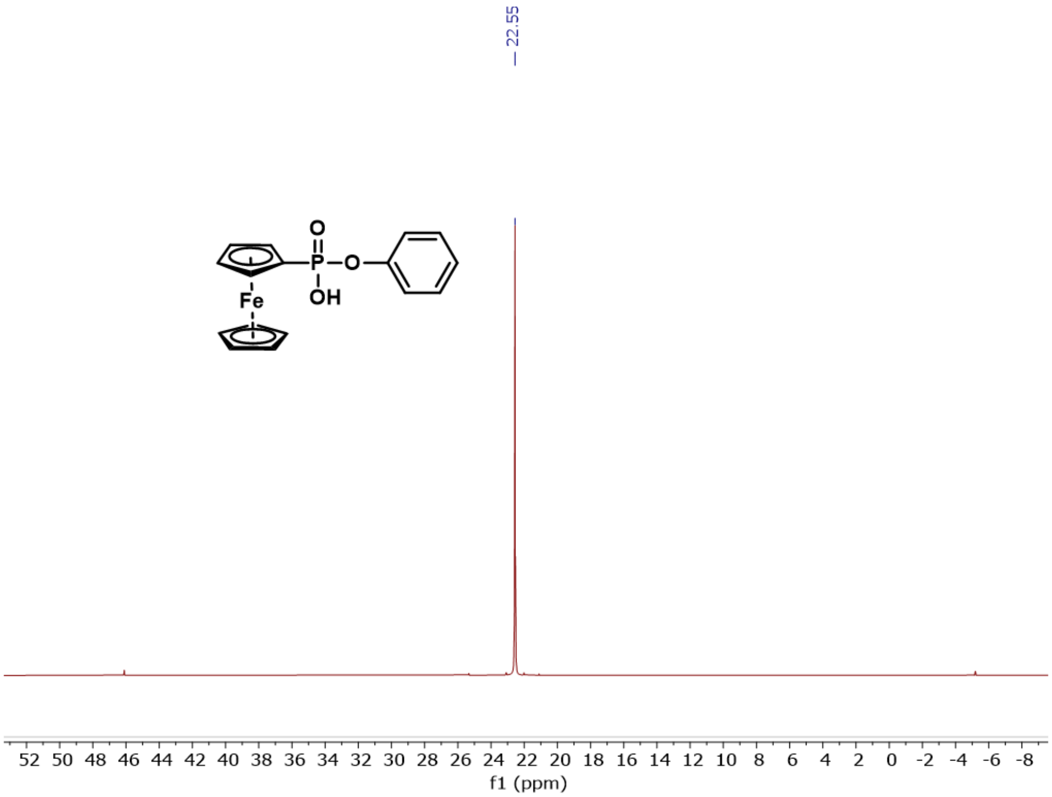

Figure S44. ^{31}P NMR spectrum acquired in CD_3OD of ferrocenyl (phenyl)phosphonic acid (**1c**).

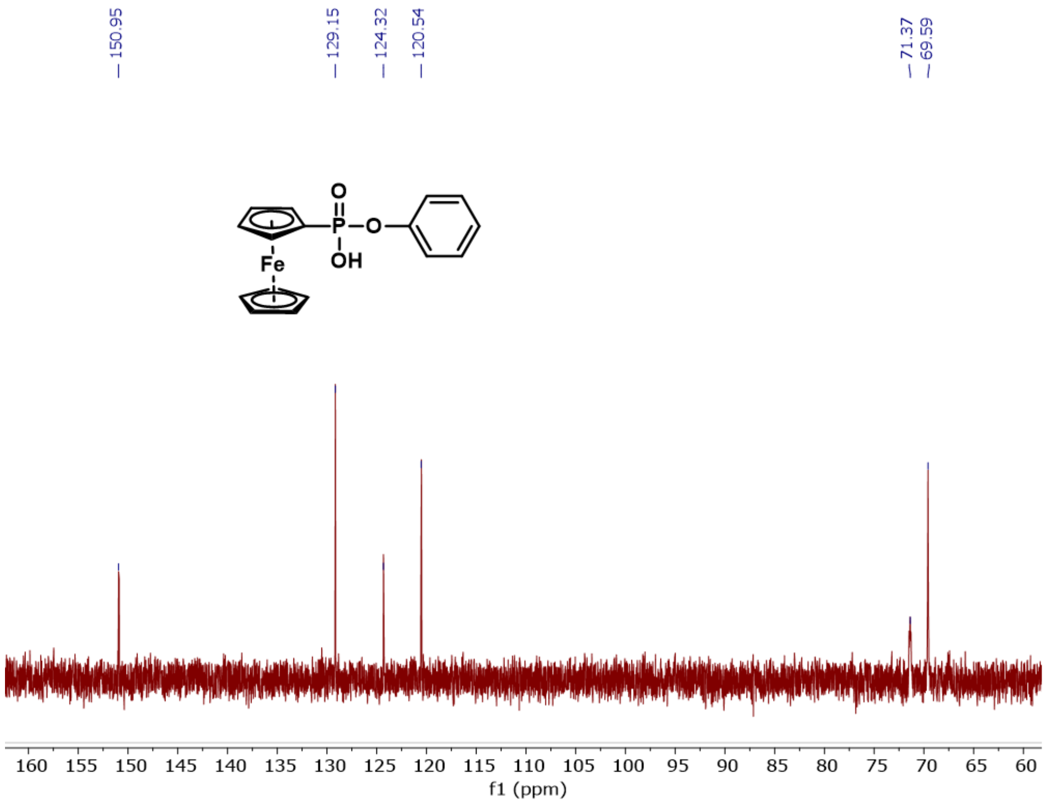

Figure S45. ^{13}C NMR spectrum acquired in CD_3OD of ferrocenyl (phenyl)phosphonic acid (**1c**).

References

[1]Oms, O.; Maurel, F.; Carré, F.; Le Bideau, J.; Vioux, A.; Leclercq, D. Improved synthesis of diethyl ferrocenylphosphonate, crystal structure of $(FcPO_3Et_2)_2$•$ZnCl_2$, and electrochemistry of ferrocenylphosphonates, $FcP(O)(OR)_2$, $FcCH_2P(O)(OR)_2$, 1,1'-fc$[P(O)(OR)_2]_2$ and $[FcP(O)(OEt)_2]_2$•$ZnCl_2$ (Fc = $(\eta^5C_5H_5)Fe(\eta^5C_5H_4)$, fc = $(\eta^5C_54_5)Fe(\eta^5C_5H_4)$, R = Et, H). *J. Organomet. Chem.* **2004**, *689*, 2654.

[2]Shekurov, P. R.; Miluykov, V. A.; Islamov, D. R.; Krivolapov, D. B.; Kataeva, O. N.; Gerasimova, T. P.; Katsyuba, S. A.; Nasybullina, G. R.; Yanilkin, V. V.; Sinyashin, O. G. Synthesis and structure of ferrocenylphosphinic acids. *J. Organomet. Chem.* **2014**, *766*, 40.

[3]Delcroix, D.; Martín-Vaca, B.; Bourissou, D.; and Navarro, D. Ring-Opening Polymerization of Trimethylene Carbonate Catalyzed by Methanesulfonic Acid: Activated Monomer versus Active Chain End Mechanisms. *Macromolecules*, **2010**, 43, 8828.

[4]Reid, L. M.; Li, T.; Cao, Y.; Berlinguette, C. P. Organic chemistry at anodes and photoanodes. *Sustain. Energy Fuels* **2018**, *2*, 1905.

[5]Warren, J. J.; Tronic, T. A.; Mayer, J. M. Thermochemistry of Proton-Coupled Electron Transfer Reagents. *Chem. Rev.* **2010**, *110*, 6961.

[6]Wiedner, E. S.; Chambers, M. B.; Pitman, C. L.; Bullock, R. M.; Miller, A. J. M.; Appel, A. M. Thermodynamic Hydricity of Transition Metal Hydrides. *Chem. Rev.* **2016**, *116*, 8655.

[7]Hu, Y.; Shaw, A. P.; Estes, D. P.; Norton, J. R. Transition-Metal Hydride Radical Cations. *Chem. Rev.* **2016**, *116*, 8462.

[8]Wu, A.; Masland, J.; Swartz, R. D.; Kaminsky, W.; Mayer, J. M. Synthesis and Characterization of Ruthenium Bis(β-diketonato) Pyridine-Imidazole Complexes for Hydrogen Atom Transfer. *Inorg. Chem.* **2007**, *46*, 11190.

[9]McLoughlin, E. A.; Waldie, K. M.; Ramakrishnan, S.; Waymouth, R. M. Protonation of a Cobalt Phenylazopyridine Complex at the Ligand Yields a Proton, Hydride, and Hydrogen Atom Transfer Reagent. *J. Am. Chem. Soc.* **2018**, *140*, 13233.
